# Visceral adipose tissue but not subcutaneous adipose tissue is associated with urine and serum metabolites

**DOI:** 10.1371/journal.pone.0175133

**Published:** 2017-04-12

**Authors:** Inga Schlecht, Wolfram Gronwald, Gundula Behrens, Sebastian E. Baumeister, Johannes Hertel, Jochen Hochrein, Helena U. Zacharias, Beate Fischer, Peter J. Oefner, Michael F. Leitzmann

**Affiliations:** 1 Department of Epidemiology and Preventive Medicine, University of Regensburg, Regensburg, Germany; 2 Institute of Functional Genomics, University of Regensburg, Regensburg, Germany; 3 Institute for Community Medicine, University Medicine Greifswald, Greifswald, Germany; 4 German Center for Neurodegenerative Diseases (DZNE) Rostock/Greifswald, Greifswald, Germany; Instituto de Investigacion Sanitaria INCLIVA, SPAIN

## Abstract

Obesity is a complex multifactorial phenotype that influences several metabolic pathways. Yet, few studies have examined the relations of different body fat compartments to urinary and serum metabolites. Anthropometric phenotypes (visceral adipose tissue (VAT), subcutaneous adipose tissue (SAT), the ratio between VAT and SAT (VSR), body mass index (BMI), waist circumference (WC)) and urinary and serum metabolite concentrations measured by nuclear magnetic resonance spectroscopy were measured in a population-based sample of 228 healthy adults. Multivariable linear and logistic regression models, corrected for multiple testing using the false discovery rate, were used to associate anthropometric phenotypes with metabolites. We adjusted for potential confounding variables: age, sex, smoking, physical activity, menopausal status, estimated glomerular filtration rate (eGFR), urinary glucose, and fasting status. In a fully adjusted logistic regression model dichotomized for the absence or presence of quantifiable metabolite amounts, VAT, BMI and WC were inversely related to urinary choline (ß = -0.18, p = 2.73*10^−3^), glycolic acid (ß = -0.20, 0.02), and guanidinoacetic acid (ß = -0.12, p = 0.04), and positively related to ethanolamine (ß = 0.18, p = 0.02) and dimethylamine (ß = 0.32, p = 0.02). BMI and WC were additionally inversely related to urinary glutamine and lactic acid. Moreover, WC was inversely associated with the detection of serine. VAT, but none of the other anthropometric parameters, was related to serum essential amino acids, such as valine, isoleucine, and phenylalanine among men. Compared to other adiposity measures, VAT demonstrated the strongest and most significant relations to urinary and serum metabolites. The distinct relations of VAT, SAT, VSR, BMI, and WC to metabolites emphasize the importance of accurately differentiating between body fat compartments when evaluating the potential role of metabolic regulation in the development of obesity-related diseases, such as insulin resistance, type 2 diabetes, and cardiovascular disease.

## Introduction

Obesity is a strong correlate of diabetes mellitus, hypertension, stroke, and several types of cancer [1–3]. The molecular mechanisms that link obesity with the development of these chronic conditions remain largely unknown. Previous epidemiological studies that examined the association between obesity and obesity-associated diseases have focused on individual molecular mediators, such as inflammatory cytokines [4]. However, the traditional epidemiologic approach that tests a single hypothesis and regresses one outcome variable on one exposure variable has limitations. Most importantly, it highlights one exposure-outcome relation while neglecting the biological complexity and the multifactorial origin of most chronic diseases [5–7]. Thus, a more comprehensive approach to study a whole biological system rather than a single risk factor is needed. Metabolomics simultaneously examines many substrates and products of the metabolism to detect metabolic alterations that are linked to human behavior, such as diet and physical activity [8–10], that are directly linked to obesity [11–14]. Thus, metabolomics offers the potential to unravel the biological mechanisms and pathways through which obesity-related phenotypes are linked to metabolism.

Better understanding the molecular mechanisms would offer the potential to develop tailored strategies for the prevention and treatment of obesity-associated chronic diseases [15, 16]. Previous studies reported dysregulation of serum and urinary metabolites, including branched chained amino acids (BCAA), amino acid-related metabolites, carnitine, phosphatidylcholines, and metabolite ratios of BCAAs to glucogenic amino acids in obesity or obesity-associated diseases [17–20]. Little is known about the relation between different adipose tissue compartments such as visceral adipose tissue (VAT), abdominal subcutaneous adipose tissue (SAT), the ratio between VAT and SAT (VSR) and metabolic profiles, and whether the contribution of those parameters are different regarding their metabolic regulation compared to body mass index (BMI) or waist circumference (WC). This is important because VAT has been considered to have multiple endocrine, metabolic, and immunological functions and to be more strongly related to obesity-associated metabolic dysregulation than SAT [21–23], which has been proposed to reduce the risk of adiposity-related metabolic dysregulation [24, 25]. However, data confirming these differences using accurate methods to quantify VAT and SAT are sparse and inconsistent [26]. The aim of the current study was to examine the associations of VAT, SAT, VSR, BMI, and WC with urinary and serum metabolites in healthy adults.

## Methods

### Study design and population

An age- and sex stratified sample of the population was drawn by the local population registries. German speaking participants aged between 20 and 70 years, living in Regensburg or adjacent regions were included in the study. In total, 233 agreed to participate (24.6% response). Of those, five participants were excluded from analyses due to pregnancy (n = 1) or diagnosed chronic diseases (n = 4). The study was conducted according to the guidelines laid down in the Declaration of Helsinki. All procedures involving human subjects were approved by the ethics committee of the University of Regensburg. The ethic committee of the Regensburg University specifically approved this study. Written informed consent was obtained from all participants.

### Metabolite measurements

Midstream urine specimens were collected from all participants (n = 228) of whom 14% had fasted overnight prior to urine collection, and centrifuged at 2000g for 10 minutes at 15°C. Supernatant urine was filled into 0.5 mL tubes and immediately stored at -80°C. Blood specimens were available from 88% of the participants (n = 200), of whom 11% had fasted overnight prior to blood withdrawal. Venous blood was drawn in serum tubes, which were then tilted twice and kept at room temperature for 30 minutes before they were centrifuged by 2500g for exactly 10 minutes at 15°C. Serum aliquots of 0.5 mL each were stored at -80°C. Metabolites were measured by nuclear magnetic resonance spectroscopy (NMR). Therefore, four hundred microliters of urine were mixed with 200 μL of phosphate buffer, pH 7.4, and 50 μL of D_2_O containing 0.75 (w%) 3-trimethylsilyl-2,2,3,3-tetradeuteropropionate (TSP) as internal standard.

Serum specimens (0.5 mL) were ultrafiltered at 4000xg for 60 minutes at 8°C using Millipore Amicon 10kDa-Filters, which had been prewashed once with 3 mL of distilled water by centrifugation at 4000g for 30 minutes at 22°C to remove filter preservatives. Subsequently, 400 μL of the serum ultrafiltrate were mixed with 200 μL of phosphate buffer, pH 7.4, and 50 μL of D_2_O containing 0.75 (w%) TSP. NMR experiments were carried out on a Bruker Avance III 600 MHz spectrometer employing a triple-resonance (^1^H, ^13^C ^31^P, ^2^H lock) cryogenic probe equipped with *z*-gradients and an automatic sample changer. All spectra were acquired following established protocols [27, 28].

### Targeted metabolomics approach

In the targeted approach the signals contributing to a given compound were integrated by peak-shape analysis to ensure that only the signals contributing to that compound are integrated and no neighbouring signals. Employing the Analytical Profiler module of AMIX 3.9.13 (BrukerBioSpin), 30 urine and 20 serum metabolites were quantified by integrating well-resolved signals of these metabolites in the acquired 2D ^1^H-^13^C HSQC and 1D ^1^H spectra relative to the TSP reference signal, ensuring that only non-overlapping signals were considered for obtaining quantitative values. From the obtained relative integrals, absolute concentrations were calculated employing individual peak calibration factors using the NMR quantification tool MetaboQuant [29]. All metabolites quantitated yielded concentration values at or above the individual lower limits of quantification (LLOQ) for at least 10% of the spectra. For a detailed list of the 30 urinary and 20 serum metabolites quantitated see the S1 Table and S2 Table.

### Untargeted metabolomics approach

The untargeted approach involves a large number of metabolites and does not depend upon a priori assumptions. Urinary and serum data sets were subjected to a comparable pre-processing routine, starting with equidistant binning of the 1D ^1^H spectra to compensate for slight shifts in signal positions across spectra due to small variations in sample pH, salt concentration, or temperature. The spectral regions of 9.5−0.5 ppm were evenly split into bins of 0.01 ppm employing Amix 3.9.13 (BrukerBioSpin). The region between 6.5−4.5 ppm, which contains the broad urea and water signals, was excluded. After exclusion of additional bins that had yielded in less than 90% of the spectra signals, a total of 701 urinary bins remained. In case of serum, the region corresponding to residual glycerol, which served as a filter preservative (3.81–3.76 ppm, 3.68–3.52 ppm), was excluded in addition, resulting in 678 bins. All urine metabolites were scaled and normalized to creatinine, and serum samples were scaled and normalized to the reference signal (TSP) to remove disturbing technical or biological variances. Chemical compounds in biological specimens were identified by their characteristic peak patterns and signal positions in the NMR spectra using reference spectra from the commercially available Bruker Biofluid Reference Compound Database BBIOREFCODE 2-0-3 and the Human Metabolome Database (HMDB) [30]. Confirmation of metabolite identification was achieved by spike-in experiments. In addition, concentrations of well-resolved compounds were calculated using calibration curves that had been generated from serially diluted external standards over a concentration range of 0.0012–10.0 mmol/L.

### Body composition measurements

VAT and SAT were quantified by ultrasonography using a B-mode ultrasound (Mindray DP-50) and a 3.5–5.0 MHz convex array transducer. Measurements were performed according to a strict protocol, details of which are described elsewhere [31]. Briefly, at the median line SAT was measured extending from the skin to the linea alba and VAT was measured reaching from the linea alba to the lumbar vertebra corpus. Height in centimeters and weight in kilograms were measured with two decimal places using a digital measuring station (seca285 measuring station, SECA, Hamburg, German). Height and weight were measured with the participants wearing underwear without shoes. BMI was calculated by dividing body weight (in kilograms) by height in meters squared (m^2^). WC was measured at the mid-point between the lower rib and the iliac crest using an inelastic tape (SECA measuring tape 201, SECA, Hamburg, Germany). Measurements were taken with the participant standing in an upright position.

### Measurement of covariables

Potential confounding variables including age, sex, current smoking status, estimated glomerular filtration rate (eGFR), physical activity, overnight fasting status, and menopausal status (women only) were assessed by standardized computer-assisted personal interviews. Physical activity levels were calculated from Metabolic Equivalents of Task (METs) [32] by a 24-hour physical activity recall. The eGFR was calculated from serum creatinine using the four-variable Modification of Diet in Renal Disease (MDRD4) formula [33]. Overnight fasting status (yes or no) was determined from self-reports of the participants before collecting urine and blood specimens.

### Statistical analysis

Descriptive statistics were used to present the characteristics of the study population. Targeted urinary and serum metabolomic data were log_2_ transformed to achieve normally distributed data and reduce heteroscedasticity. Pearson correlations between all quantified, log_2_-transformed metabolites were calculated and correlation coefficients were considered weak (r<0.4), moderate (r≥0.4-<0.6), or strong (r≥0.6) [34]. Differences between men and women, fasting and non-fasting participants, urinary glucose and non-glucose subjects were tested using Kruskal-Wallis and χ^2^ tests. In sensitivity analyses, effect-measure modification by age and sex interaction was examined by testing multiplicative interaction terms in regression models using likelihood ratio tests. For the detection of unbalanced regulation of compounds in untargeted data, the total spectral area for each NMR spectrum was calculated and tested for normal distribution using the Shapiro-Wilk test [35]. To reduce heteroscedasticity, data were normalized using variance stabilization with 60% of the urinary and 65% of the serum features, respectively. Identification of metabolites was further facilitated by use of the statistical correlation technique (STOCSY), which utilizes the presence of multiple collinearities in a set of spectra [36]. To this end, based on a set of ^1^H 1D spectra a pseudo 2D spectrum is computed by calculating the correlation between the intensities of the different bins. For bins corresponding to the same metabolite strong correlations are expected independent of the presence of dipolar or J couplings, which are utilized in standard 2D spectra. Therefore, based on the STOCSY technique bins belonging to the same molecule may be identified and thereby supporting metabolite identification. Note, for the computation of the STOCSY spectra were normalized to total spectral area [37].

Multiple linear regression models were used to examine associations of VAT, SAT, VSR, BMI, and WC to urinary and serum metabolites. Models were adjusted for study, age, and sex (Model 1). The fully-adjusted model added a multiplicative age-sex interaction, smoking status (former, current, never smoking), physical activity level (continuous), menopausal status (pre-, peri-, postmenopausal), fasting status (overnight fasting: yes, no), urinary glucose (elevated glucose: yes, no), and eGFR (continuous) were applied (Model 2). Stratified analyses were performed regarding sex. We tested for linearity by including squared and cubic terms of VAT, SAT, VRS, BMI and WC. To account for the considerable amount of values below the LLOQ of some metabolites (S1 Table and S2 Table), linear regression models were only calculated for those metabolites for which at least 75% of data were available. Otherwise, metabolites were dichotomized as detectable and undetectable and logistic regression models were run while adjusting for the same models 1 and 2 as described above.

Multiplicity was corrected by controlling the false discovery rate (FDR) according to Benjamini and Hochberg [38] at a level of 5%. Dependent and independent variables were standardized by subtracting the mean and dividing by the standard deviation for all regression models to assure the comparability of effect estimates. All reported p values are two-tailed, FDR-adjusted, and values <0.05 were deemed statistically significant. R statistics 3.1.2 were used for analyses.

## Results

Characteristics of the study population are presented in Table 1. The mean age of study participants was 52 years and 13.2% of the participants were fasting. Women had lower VAT (p<0.001), VSR (p<0.001), BMI (p = 0.008) and WC (p<0.001) than men. In contrast, women had a higher mean SAT (p = 0.011) than men. Differences between men and women, fasting and non-fasting participants as well as between subjects with elevated urinary glucose and subjects with no urinary glucose were found for urinary and serum metabolites (S3 Table and S4 Table).

**Table 1:** Characteristics of the study population by sex.

	Total mean (SD)	Women mean (SD)	Men mean (SD)	p-value
N	228	121	107	
Age	51.96 (12.55)	52.80 (12.00)	50.97 (13.15)	0.388
Non-fasting	198	105	93	0.511
Current smoking	31	12	19	0.075
Physical activity level*	1.67 (0.27)	1.67 (0.27)	1.68 (0.28)	0.888
VAT thickness US (cm)	6.79 (2.85)	6.29 (2.80)	7.38 (2.81)	<0.001
SAT thickness US (cm)	2.06 (0.85)	2.17 (0.89)	1.93 (0.78)	0.011
VSR	3.77 (2.14)	3.23 (1.70)	4.41 (2.41)	<0.001
BMI (kg/m^2^)	26.61 (4.66)	25.97 (4.99)	27.36 (4.13)	0.008
Waist circumference (cm)	91.16 (13.56)	85.53 (11.99)	97.75 (12.30)	<0.001

Entries are mean (standard deviation) for continuous variables and absolute numbers for categorical variables. VAT = visceral adipose tissue, SAT = subcutaneous adipose tissue, BMI = body mass index, US = ultrasonography, n = 228. p-value from Kruskal-Wallis test.

*Calculated from Metabolic Equivalents of Task.

### Results from targeted metabolomics approach

Pearson correlation analyses to assess correlations between metabolites showed moderate (r≥0.4) and strong (r≥0.6) correlations between 36% of the urinary metabolites and 44% of the serum metabolites (S1 Fig and S2 Fig).

Results from regression models to examine relations of obesity measures to urinary metabolites in the overall population revealed significant inverse and positive relations of VAT, BMI, and WC to the detection of urinary metabolites in age and sex adjusted models (Model 1) and in fully adjusted models considering further adjustments for smoking, menopause status, physical activity, urinary glucose, and eGFR (Model 2) (Table 2). Specifically, VAT was inversely related to the detection of choline (ß = -0.18, p = 2.73*10^−3^), guanidinoacetic acid (ß = -0.12, p = 0.04), and glycolic acid (ß = -0.20, 0.02), and positively related to the detection of ethanolamine (ß = 0.18, p = 0.02) and dimethylamine (ß = 0.32, p = 0.02) in fully adjusted models (Model 2). Note that the obtained choline concentrations were most likely influenced by related molecules due to the presence of partial overlap in the analyzed NMR spectra. Further, BMI and WC were additionally inversely related to the detection of glutamine and lactic acid in fully adjusted models (Model 2). Moreover, WC was inversely associated with the detection of serine in fully adjusted models (Model 2). No significant associations were found between SAT or VSR and urinary metabolites in any of the analyses.

**Table 2:** Significant associations between measures of obesity and urinary metabolite levels.

	Metabolite	Model 1			Model 2	
		ß		p	ß	p
VAT	Formic acid*		-0.09	4.81*10^−4^	-0.07	0.19
	Choline		-0.12	5.26*10^−4^	-0.18	2.73*10^−3^
	Methanol		0.23	0.01	0.10	0.29
	Ethanolamine		0.15	0.03	0.18	0.02
	Dimethylamine		0.23	0.01	0.32	0.02
	Guanidinoacetic acid		-0.14	0.03	-0.12	0.04
	Glycolic acid		-0.16	0.03	-0.20	0.02
BMI	Formic acid*		-0.04	0.03	-0.04	0.20
	Alanine		0.10	0.01	0.17	0.01
	Betaine		-0.10	0.01	-0.13	0.03
	Choline		-0.11	0.01	-0.17	0.01
	Creatine		-0.10	0.01	-0.10	0.10
	Dimethylamine		0.18	5.51*10^−4^	0.26	0.01
	Ethanolamine		0.13	2.10*10^−3^	0.15	0.02
	Glutamine		-0.12	3.32*10^−3^	-0.16	0.01
	Glycolic acid		-0.15	6.74*10^−4^	-0.19	0.01
	Guanidinoacetic acid		-0.14	1.30*10^−3^	-0.17	0.01
	Lactic acid		-0.08	0.02	-0.16	0.01
	Methanol		0.12	0.01	0.05	0.36
	Taurine		-0.08	0.03	-0.10	0.10
WC	Formic acid*		-0.02	0.01	-0.02	0.21
	Alanine		0.05	1.93*10^−3^	0.07	0.05
	Betaine		-0.05	1.67*10^−3^	-0.05	0.12
	Choline		-0.05	1.67*10^−3^	-0.08	3.99*10^−3^
	Creatine		-0.04	0.01	-0.03	0.13
	Dimethylamine		0.07	7.71*10^−5^	0.09	3.99*10^−3^
	Ethanolamine		0.06	6.87*10^−4^	0.06	0.01
	Glutamine		-0.06	8.49*10^−4^	-0.06	0.01
	Glycolic acid		-0.05	1.67*10^−3^	-0.06	0.01
	Guanidinoacetic acid		-0.06	6.87*10^−4^	-0.07	0.01
	Lactic acid		-0.03	0.02	-0.06	0.01
	Methanol		0.03	0.04	0.02	0.30
	Phenylalanine		-0.04	0.03	-0.02	0.28
	Serine		-0.04	0.02	-0.05	0.02
	Taurine		-0.03	0.02	-0.04	0.10

Model 1: regression model adjusted for study, age (non-linear) and sex. Model 2: regression model adjusted for study, age and sex interaction (non-linear), smoking status, menopausal status (women only), physical activity, urinary glucose, and eGFR. VAT = visceral adipose tissue, BMI = body mass index, WC = waist circumference, ß = beta coefficient, p-value = corrected for multiple testing by controlling the false discovery rate.

*linear model applied, p-values<0.05 after correction for multiple testing were considered significant.

After stratifying by sex, results showed inverse relations of VAT to the detection of choline (ß = -0.18; p = 2.73*10^−3^), glycolic acid (ß = -0.58, p = 0.04), guanidinoacetic acid (ß = -0.36, p = 0.04), lactic acid (ß = -0.32, p = 0.04), and serine (ß = -0.41, p = 0.04) and positive relations to the detection of ethanolamine (ß = 0.71, p = 0.04) and dimethylamine (ß = 0.65, p = 0.04) in fully adjusted models among men (S5 Table). By comparison, BMI and WC were additionally inversely associated with the detection of betaine and glutamine in fully adjusted models. No relation was found between BMI and serine in fully adjusted models among men. Among women, VAT was positively related with the detection of dimethylamine (ß = 0.12; p = 0.04) in the fully adjusted model. BMI was significantly inversely related to the detection of creatine and glycolic acid and positively related to the detection of dimethylamine and methanol in the age and sex adjusted models. However, after full adjustment, estimated relations were attenuated and associations of BMI with the detection of urinary metabolites were no longer statistically significant. WC was positively associated with the detection of dimethylamine in fully adjusted models (Model 2) among women. No significant relations were found between SAT or VSR and urinary metabolites among men or women.

When regression models were applied to serum metabolites, results showed no significant results between VAT, SAT, VSR, BMI, and WC and concentrations of serum metabolites for the overall population and among subgroups. Also, no significant results were found between the highest and lowest quartiles of VAT, SAT, VSR, BMI, or WC and serum metabolites in the overall population or among subgroups.

### Results from untargeted metabolomics approach

Multiple linear regression models to detect relations of VAT and SAT to the urinary metabolic fingerprints in the overall population showed statistically significant associations between VAT and 38 urinary bins in fully adjusted models (Model 2) (Table 3). Metabolite identification was performed by comparison with reference spectra of pure compounds. The metabolite assignments were further corroborated by means of an STOCSY analysis (S3 Fig). The STOCSY helps identifying signals belonging to the same metabolite by means of a correlation analysis. In its appearance, it is comparable to a conventional 2D TOCSY experiment. In this representation, positive and negative correlations are depicted in red and blue, respectively. Correlations above 0.50 respectively below -0.50 are shown. As the plot of the STOCSY is fairly crowded individual metabolites were analyzed by employing the 1D version of the STOCSY algorithm. In case that more than one compound contributed to a given bin, all corresponding molecules are indicated in Table 3. Positive relations were found to lysine, dimethylamine and tyrosine, while negative relations were found to 4-hydroxyhippuric acid, acetone, citric acid, glycine, L-pyroglutamic acid, methylmalonic acid, scyllo-inositol, and formic acid. In addition, significant associations were found between BMI and 35 bins and between WC and 62 bins (data not tabulated). Most bins that were significantly associated with VAT were also found to be significant in models with BMI and WC. However, VAT but none of the other parameters was inversely related to 4-hydroxyhippuric acid in the overall study population in fully adjusted models (Model 2). By comparison, no significant relations were found between SAT or VSR and urinary bins.

**Table 3:** Significant results from multiple regression analyses on the relation of VAT to urinary bins (all subjects).

		Model 1		Model 2		
Anthropometric variable	bin (ppm)	ß	p	ß	p	metabolite identification
VAT	3.795	0.0341	0.0106	0.0388	0.0155	Lysine
	1.485	0.0981	0.0500	0.0918	0.0285	
	2.435	-0.0354	0.0316	-0.0329	0.0318	3-Hydroxy-3-methylglutaric acid, others
	2.475	-0.0334	0.0695	-0.0355	0.0102	
	3.945	-0.0452	0.0695	-0.0475	0.0320	4-Hydroxyhippuric acid
	6.955	-0.0717	0.0277	-0.0860	0.0100	
	6.965	-0.0803	0.0277	-0.0843	0.0227	
	2.235	-0.0480	0.0562	-0.0526	0.0318	Acetone
	2.725	0.0518	0.0546	0.0601	0.0304	Dimethylamine
	3.205	-0.0324	0.0546	-0.0458	0.0097	Choline, others
	3.525	-0.0455	0.0562	-0.0463	0.0344	
	4.065	-0.1101	0.0546	-0.1337	0.0175	
	2.675	-0.1161	0.0546	-0.1358	0.0304	Citric acid
	2.735	0.0633	0.0772	0.0813	0.0304	Dimethylamine
	3.565	-0.0661	0.0546	-0.0669	0.0349	Glycine, others
	2.405	-0.0540	0.0277	-0.0583	0.0100	L-Pyroglutamic acid
	2.515	-0.0430	0.0313	-0.0430	0.0297	
	2.395	-0.0283	0.1068	-0.0308	0.0465	
	2.425	-0.0318	0.0695	-0.0348	0.0320	
	1.235	-0.0428	0.0546	-0.0457	0.0318	Methylmalonic acid
	3.355	-0.0503	0.0277	-0.0561	0.0223	scyllo-Inositol
	3.055	0.0952	0.0587	0.1149	0.0304	Creatinine
	2.965	-0.0347	0.0772	-0.0394	0.0435	Asparagine, unknown
	2.975	-0.0331	0.0772	-0.0674	0.0381	
	8.455	-0.0911	0.0316	-0.1013	0.0223	Formic acid
	3.535	-0.0324	0.0695	-0.0320	0.0465	Sugar compounds, unknown
	1.355	0.0234	0.0271	0.0226	0.0392	unknown
VAT	2.325	-0.0303	0.0661	-0.0307	0.0320	unknown
	2.335	-0.0259	0.1025	-0.0276	0.0320	unknown
	2.365	-0.0362	0.0562	-0.0377	0.0320	unknown
	2.605	-0.0119	0.0346	-0.0508	0.0217	unknown
	2.615	-0.0157	0.0446	-0.0613	0.0162	unknown
	2.625	-0.0198	0.0018	-0.0511	0.0045	unknown
	2.645	-0.0468	0.0546	-0.0454	0.0449	unknown
	6.585	-0.0657	0.0277	-0.0692	0.0223	unknown
	6.785	-0.0369	0.0772	-0.0439	0.0320	unknown
	6.805	-0.0632	0.0433	-0.0807	0.0223	unknown
	6.945	-0.0384	0.1147	-0.0489	0.0320	unknown

Model 1: linear regression model adjusted for study, age (non-linear) and sex. Model 2: regression model adjusted for study, age and sex interaction (non-linear), smoking status, menopausal status (women only), physical activity, urinary glucose, and eGFR. VAT = visceral adipose tissue, ß = beta coefficient, p-value = corrected for multiple testing by controlling the false discovery rate. p-values<0.05 after correction for multiple testing were considered significant.

In subgroup analyses stratified by gender, no significant relations were found between VAT, SAT, VSR, BMI, or WC and urinary bins. However, when analyses were restricted to non-fasting participants, significant associations were found between VAT and 80 bins in fully adjusted models (Model 2) (S6 Table). No relations were found between SAT or VSR and urinary bins. By comparison, BMI and WC were significantly related to 99 and 70 bins, respectively (data not tabulated). Significant results from analyses among all subjects were reproduced in sensitivity analyses among non-fasting subjects. In addition, VAT, BMI, and WC were inversely associated with acetone, choline, citric acid, asparagine/unknown, and L-pyroglutamic acid in this group. Significant associations were found between VAT, BMI, and WC to urinary bins in analyses restricted to subjects with no urinary glucose in fully adjusted models (Model 2). Specifically, VAT was related to 11 bins among subjects with no urinary glucose (S6 Table). These bins were also found to be significant in the overall study population (e.g., 4-hydroxyhippuric acid or L-pyroglutamic acid).

Linear regression models to detect relations of VAT and SAT to serum bins showed no significant results in the overall population. VSR, BMI, and WC also showed no significant relations to individual serum bins. When analyses were stratified by gender, significant results were found between VAT and 20 serum bins among men in fully adjusted models (Model 2) (Table 4). Of these significantly related 20 bins, twelve could be assigned to specific metabolites. VAT was positively related to citric acid and tryptophan, and showed an inverse relation to ketoleucine. In addition, positive relations were found between VAT and essential amino acids, including valine, phenylalanine, and isoleucine. Note that the bin corresponding to isoleucine shows partial overlap with ketoleucine. By comparison, among women VAT was inversely related to three serum bins in fully adjusted models (Model 2). Those bins were not related to VAT among men. However, only one of the significantly inversely related bins could be identified as lipid-cholesterol. No relations were found between SAT, VSR, BMI, or WC and serum bins among men or women.

**Table 4:** Significant results from gender specific multiple regression analyses on the relation between VAT and serum bins.

		Model 1		Model 2		
Anthropometric variable	bin (ppm)	ß	p	ß	p	metabolite identification
**Men (n = 98)**						
VAT	0.995	0.0413	0.0146	0.0588	0.0015	Valine
	0.985	0.0381	0.0476	0.0138	0.0285	
	1.035	0.0813	0.0246	0.0562	0.0128	
	0.935	0.0008	0.9952	0.0466	0.0178	Isoleucine, ketoleucine
	2.605	-0.0160	0.9746	-0.0508	0.0117	Ketoleucine
	2.615	-0.0175	0.9746	-0.0613	0.0060	
	2.625	-0.0084	0.9818	-0.0502	0.0047	
	1.065	0.0444	0.8602	0.0993	0.0006	Isobutyric acid, unknown
	1.075	0.0314	0.8602	0.0798	0.0017	
	2.655	0.0186	0.8602	0.0442	0.0093	Methionine, unknown
	7.375	0.0170	0.9746	0.0669	0.0202	Phenylalanine, unknown
	7.555	0.0485	0.9802	0.1351	0.0274	Tryptophan
	2.595	0.0203	0.9746	0.0538	0.0285	unknown
	2.635	0.0167	0.9746	0.0672	0.0003	unknown
	4.345	-0.2167	0.7141	-0.4572	0.0013	unknown
	6.745	0.4734	0.8602	0.7706	0.0351	unknown
	7.485	-0.2613	0.8602	-0.4372	0.0388	unknown
	1.085	0.0271	0.8602	0.0638	0.0202	unknown
	1.105	0.0102	0.9818	0.0826	0.0161	unknown
	7.675	-0.2390	0.7141	-0.3350	0.0082	unknown
**Women (n = 102)**						
VAT	0.735	-0.0242	0.5579	-0.0482	0.0136	Lipid-cholesterol
	0.605	-0.4264	0.1599	-0.3640	0.0136	unknown
	7.845	-0.2337	0.1888	-0.4020	0.0368	unknown

Model 1: linear regression model adjusted for study, age (non-linear) and sex. Model 2: linear regression model adjusted for study, age and sex interaction (non-linear), smoking status, menopausal status (women only), physical activity, urinary glucose, and eGFR. In each case, the stratification variable was excluded from the model. VAT = visceral adipose tissue, ß = beta coefficient, p-value = corrected for multiple testing by controlling the false discovery rate. p-values<0.05 after correction for multiple testing were considered significant.

## Discussion

### Discussion of urine results

Results from the targeted metabolomics approach showed significant positive and inverse relations of VAT, BMI, and WC to urinary metabolites in the overall study population and in stratified analyses among men and women. Specifically, VAT, BMI, and WC were consistently inversely related to the detection of choline in the overall study population and in stratified subgroups including men. The strongest and most significant associations were found between VAT and choline. This indicates that predominantly VAT is related to choline metabolism. Choline is an essential nutrient, playing a complex role in human metabolism [39]. Specifically, choline is needed for neurotransmitter synthesis (acetylcholine), cell-membrane signaling (phospholipids), lipid transport (lipoproteins), and methyl-group metabolism (homocysteine reduction) [40]. Previous metabolomics studies reported inverse relations of serum choline to type 2 diabetes [41] and cardiovascular disease [42]. A lack of choline can lead to a fatty liver condition due to lack of very low density lipoprotein (VLDL), which shuttles lipids away from the liver [39]. In addition, choline deficiency is associated with increased levels of several inflammatory markers that link obesity to obesity-associated diseases, including CRP, homocysteine, IL-6, and TNF [39, 43]. A possible mechanism is that with inadequate choline stores, the capacity to methylate homocysteine to methionine is diminished and consequently, plasma levels of homocysteine increase [39]. Homocysteine was demonstrated to contribute to the initiation and progression of vascular disease by activating monocytes, resulting in the secretion of cytokines that amplify the inflammatory response [44].

In addition, VAT, BMI, and WC were positively related to dimethylamine in the overall population, among men and women. Compared to other measures of obesity, the strongest relations were found between VAT and dimethylamine and only weak relations were found between WC and dimethylamine. This indicates that VAT may be the main indicator for increased dimethylamine levels. A positive association of BMI with urinary dimethylamine was reported recently by one study among adults of the US population and was replicated among British adults [45]. Dimethylamine is a gut-microbial metabolite related to the gut microbial metabolism of choline. The impact of the gut microbiota on dietary choline, for the most part originating from carnitine and lecithin, to produce trimethylamine by choline trimethylamine-lyases and the subsequent conversion of trimethylamine and trimethylamine-N-oxide to dimethylamine describes a pathway that has been linked to the development of atherosclerosis and cardiovascular disease [46, 47]. Increased urinary excretion of microbial metabolites from co-metabolic processing of choline has also been related to steatosis, fatty liver, and insulin resistance in animals fed high-fat diets [48]. In humans, an increase in energy intake from high-fat diets rapidly influences the composition of the gut microbiota [49]. These compositional changes in microbiota may lead to increases in systemic endotoxin levels [50] that consequently may contribute to low-grade inflammation, insulin resistance, adipocyte hyperplasia, and decreased β-cell function, all of which characterize the metabolic syndrome [49].

Results from the untargeted metabolomics approach showed that VAT, BMI, and WC were consistently related to numerous urinary bins in the overall study population and in stratified subgroups. No relations were found between SAT or VSR and urinary bins. With the application of the untargeted metabolomics approach in combination with multiple linear regression models, candidate metabolites were identified that were not included in the targeted metabolomics software to quantify metabolite concentrations. Specifically, VAT was inversely related to numerous urinary bins identified as 4-hydroxyhippuric acid, acetone, L-pyroglutamic acid, citric acid, and scyllo-inositol. Moreover, VAT was positively related to lysine and creatinine. Notably, VAT but none of the other adiposity measures was significantly inversely related to 4-hydroxyhippuric acid. As a glycine conjugate of 4-hydroxybenzoic acid, 4-hydroxyhippuric acid is an end-product derived of polyphenol metabolism by the intestinal microflora [51]. A previous study suggested that the intake of fruits containing polyphenols rich in anthocyanins (i.e., berries and grapes) may increase 4-hydroxyhippuric acid in the urine [52]. Anthocyanins are naturally occurring polyphenols with anti-inflammatory activity [53, 54]. Hence, the observed inverse relations of VAT to 4-hydroxyhippuric acid found in the present study may reflect a diet lacking in health benefitting nutrients among participants with increased VAT. However, concentrations of 4-hydroxyhippuric acid were not quantitated in the present study. Nevertheless, 4-hydroxyhippuric acid may be a promising candidate metabolite for future obesity research.

### Discussion of serum results

No significant relations were found between VAT, SAT, VSR, BMI, or WC and quantified serum metabolites. There are several possible explanations that might explain why we found significant associations of adiposity measures with urinary metabolites but not with serum metabolites. Although the human urine metabolome is generally believed to constitute a subset of the human serum metabolome [55], a few hundred compounds identified in urine still await their detection in blood [56]. The fact that so many compounds seem to be unique to urine most likely reflects the ability of the kidney to concentrate certain metabolites from blood, with urinary levels of some metabolites exceeding those in serum more than a 1000-fold (e.g., histamine) [56]. In addition, the use of ultrafiltration in sample pretreatment allows only measurement of the protein-unbound fraction of metabolites [57]. As a result, many serum metabolites may go undetected. This holds particularly true for NMR spectroscopy, a fairly insensitive method with lower limits of quantification in the low to medium micromolar range.

In the untargeted analyses, VAT but none of the other measures of obesity was related to serum bins which was evident in sex-stratified analyses only. This indicates a possible interaction of sex on the association between VAT and serum metabolites that needs further investigation. Two recent studies demonstrated major differences in metabolic fingerprints between men and women [58, 59]. Specifically, the authors reported that the serum metabolic profile showed no significant difference between lean and obese participants in the overall study population. However, similar to the present study, they reported significantly altered metabolites among men, whereas no significant differences were observed among women [59]. Interestingly, no relations were found between SAT, VSR, BMI, or WC and serum metabolites, indicating that VAT may be predominantly responsible for metabolite alterations in serum among men. Note that the significant relations found between VAT and serum bins were detected in untargeted analyses only. However, results from targeted analyses showed that the association between VAT and valine (ß = 0.07; p = 0.06) and VAT and isoleucine (ß = 0.10; p = 0.05) were “borderline significant” after the correction for multiple testing in fully adjusted models (Model 2). These differences may be explained by the fact that in targeted analyses only those integrals are considered that were above the LLOQ, whereas in untargeted analyses all integrals for one bin were considered. Moreover, the additional normalization of spectral areas using VSN on untargeted data may have attenuated some of the relations. Finally, because of differences in the distribution between targeted and untargeted data, different statistical analyses were performed that resulted in differences in significance and strengths of the relations between anthropometric parameters and metabolites.

The positive relations found between VAT and serum valine and isoleucine are consistent with findings from previous studies that reported significantly higher levels of BCAA, including isoleucine and valine in obese participants compared to lean participants [14, 59, 60]. Moreover, plasma BCAA levels were positively correlated with insulin resistance in a previous study [14]. Another study concluded, that BCAAs and aromatic amino acids could serve as predictors of the future development of diabetes [61]. No relations of SAT, VSR, BMI, or WC to serum BCAAs were found in the present study, indicating that VAT is predominantly involved in BCAA metabolism. This supports findings from previous studies that identified adipose tissue and adipocytes to be a main contributor to whole-body, branched-chain amino acid catabolism [62]. Leucine, isoleucine, and valine are transported into adipocytes and metabolized in the mitochondria to form the anapleurotic intermediates acetyl-CoA and succinyl-CoA, thereby enabling maximal pyruvate metabolism to citrate and subsequent lipogenesis [63]. Further, it has been shown that adipocytes readily metabolize BCAAs to lipogenic precursors and that the maximal lipogenic rate requires replenishment of the tricarboxylic acid (TCA) cycle [62].

### Strengths and limitations

The major strength of our study is that it represents, to the best of our knowledge, the first cross-sectional study to examine relations of VAT and SAT assessed by ultrasound to NMR-measured urinary and serum metabolites. Although gold standard methods to measure VAT and SAT are magnetic resonance imaging (MRI) or computer tomography (CT), these approaches are limited in field conditions due to the associated costs and issues regarding accessibility, and in terms of CT potential adverse effects of radiation. We recently reported that ultrasound represents a suitable technique to assess VAT and SAT accurately and reproducibly in epidemiologic research settings [31]. A further asset of our study is that we considered several different body fat measures and conducted informative stratified analyses. However, our study has several limitations that need to be considered when interpreting the results. First, our analyses were based on a single laboratory measurement and they may not represent the true long-term average urinary and serum concentrations of metabolites. However, a previous study on reliability of metabolite concentrations over time stated that for most metabolites a single measurement may be sufficient [64]. Potential limitations of our study include the relatively small sample size, potentially resulting in insufficient statistical power to detect relations, particularly in subgroup analyses. In addition, the cross-sectional nature of our study design precludes an assessment of temporality and reverse causality. Finally, the majority of our urine and blood specimens were non-fasting and we lacked sufficient power to conduct analyses among fasting participants.

### Conclusion

In conclusion, we found VAT to be consistently related to urinary and serum metabolites. By comparison, SAT and VSR showed no relation to urinary or serum metabolites, further indicating that VAT is a major contributor to metabolite regulation. In addition, relations between VAT and serum metabolites occurred in a sex-dependent manner. The present findings suggest potential pathways that may explain possible mechanisms underlying obesity-associated diseases. Specifically, metabolites and their related pathways could be identified that may explain mechanisms of obesity-induced low-grade inflammation. In addition, the accumulation of VAT may be, at least in part, responsible for dysregulation of BCAAs in serum. BMI and WC were consistently related to urinary metabolites but no relations were found between BMI or WC and serum metabolites. Compared to the relations of WC and BMI to urinary metabolites, relations between VAT and metabolites were generally stronger. In addition, some relations of metabolites including 4-hydroxyhippuric acid were unique to VAT. Nevertheless, the number of metabolites that were significantly associated with VAT was smaller than the number of metabolites related to BMI and WC. This indicates that the impact of VAT, BMI, and WC differs regarding metabolic regulation and metabolic pathways associated with VAT cannot precisely be reflected by BMI or WC alone. The distinct relations of VAT, BMI and WC and the null findings of SAT and VSR to metabolites emphasize the importance of accurately differentiating between body fat compartments when evaluating the potential role of adiposity-associated metabolic regulation in the development of obesity-related diseases.

## Supporting information

S1 Fig Pearson correlation matrix between creatinine normalized urinary metabolite concentrations. (JPG)

S2 Fig Pearson correlation matrix between serum metabolite concentrations. (JPG)

S3 Fig STOCSY two-dimensional representation of the urinary ^1^H NMR spectra. Positive and negative correlations are depicted in red and blue, respectively. Correlations above 0.50 respectively below -0.50 are shown. The water region and the region of the broad urea signal between 6.5–4.5 ppm was excluded.
(JPG)

S1 Table Gender-dependent urinary metabolite concentrations. All metabolites were quantified by 1D or 2D nuclear magnetic resonance spectroscopy: ^1^Metabolites quantified from 1D spectra, ^2^Metabolites quantified from 2D spectra, conc. = concentration, min = minimum, max = maximum, N = number of values above LLOQ. *Concentrations are influenced by Phenylalanine, Phenylacetylglycine und Phenylacetylglutamine. ^+^Choline concentrations are likely to be influenced by other compounds including creatinine.
(DOCX)

S2 Table Gender-dependent serum metabolite concentration. All metabolites were quantified by 1D or 2D nuclear magnetic resonance spectroscopy: ^1^Metabolites quantified from 1D spectra, ^2^Metabolites quantified from 2D spectra, conc. = concentration, min = minimum, max = maximum, N = number of available values.
(DOCX)

S3 Table Urinary metabolite interaction with sex, fasting status, and urinary glucose. P-value from Kruskal-Wallis test.
(DOCX)

S4 Table Serum metabolite interaction with sex, fasting status, and urinary glucose. P-value from Kruskal-Wallis test.
(DOCX)

S5 Table Significant associations between measures of obesity and urinary metabolite levels among subgroups. Model 1: regression model adjusted for study, age (non-linear) and sex. Model 2: regression model adjusted for study, age and sex interaction (non-linear), smoking status, menopausal status (women only), physical activity, urinary glucose, and eGFR. VAT = visceral adipose tissue, BMI = body mass index, WC = waist circumference, ß = beta coefficient, p-value = corrected for multiple testing by controlling the false discovery rate. *linear model applied, p-values<0.05 after correction for multiple testing were considered significant.
(DOCX)

S6 Table Significant results from regression analyses to detect relations of VAT to urinary bins among subgroups. Model 1: linear regression model adjusted for study, age (non-linear) and sex. Model 2: linear regression model adjusted for study, age and sex interaction (non-linear), smoking status, menopausal status (women only), physical activity, urinary glucose, and eGFR. In each case, the stratification variable was excluded from the model. VAT = visceral adipose tissue, ß = beta coefficient, p-value = corrected for multiple testing by controlling the false discovery rate. p-values<0.05 after correction for multiple testing were considered significant.
(DOCX)

S7 Table Significant results from multiple regression analyses on the relation between VAT and serum bins among subgroups. Model 1: linear regression model adjusted for study, age (non-linear) and sex. Model 2: linear regression model adjusted for study, age and sex interaction (non-linear), smoking status, menopausal status (women only), physical activity, urinary glucose, and eGFR. In each case, the stratification variable was excluded from the model.
VAT = visceral adipose tissue, ß = beta coefficient, p-value = corrected for multiple testing by controlling the false discovery rate. p-values<0.05 after correction for multiple testing were considered significant.
(DOCX)

## References

[1] Ross R, Bradshaw AJ. The future of obesity reduction: beyond weight loss. Nat Rev Endocrinol. 2009;5(6):319–25. Epub 2009/05/08. doi: 10.1038/nrendo.2009.78 19421242 10.1038/nrendo.2009.78

[2] Bastard JP, Maachi M, Lagathu C, Kim MJ, Caron M, Vidal H, et al Recent advances in the relationship between obesity, inflammation, and insulin resistance. European cytokine network. 2006;17(1):4–12. 16613757

[3] Renehan AG, Tyson M, Egger M, Heller RF, Zwahlen M. Body-mass index and incidence of cancer: a systematic review and meta-analysis of prospective observational studies. Lancet. 2008;371(9612):569–78. doi: 10.1016/S0140-6736(08)60269-X 18280327 10.1016/S0140-6736(08)60269-X

[4] Jung UJ, Choi M-S. Obesity and Its Metabolic Complications: The Role of Adipokines and the Relationship between Obesity, Inflammation, Insulin Resistance, Dyslipidemia and Nonalcoholic Fatty Liver Disease. International Journal of Molecular Sciences. 2014;15(4):6184–223. doi: 10.3390/ijms15046184 24733068 10.3390/ijms15046184 PMC4013623

[5] Hu FB. Metabolic profiling of diabetes: from black-box epidemiology to systems epidemiology. Clinical chemistry. 2011;57(9):1224–6. doi: 10.1373/clinchem.2011.167056 21690202 10.1373/clinchem.2011.167056

[6] Lund E, Dumeaux V. Systems epidemiology in cancer. Cancer epidemiology, biomarkers & prevention: a publication of the American Association for Cancer Research, cosponsored by the American Society of Preventive Oncology. 2008;17(11):2954–7. 10.1158/1055-9965.EPI-08-0519 18990736

[7] Muller G. Personalized prognosis and diagnosis of type 2 diabetes—vision or fiction? Pharmacology. 2010;85(3):168–87. doi: 10.1159/000283780 20173359 10.1159/000283780

[8] Gibney MJ, Walsh M, Brennan L, Roche HM, German B, van Ommen B. Metabolomics in human nutrition: opportunities and challenges. Am J Clin Nutr. 2005;82(3):497–503. 16155259 10.1093/ajcn.82.3.497

[9] Primrose S, Draper J, Elsom R, Kirkpatrick V, Mathers JC, Seal C, et al Metabolomics and human nutrition. Br J Nutr. 2011;105(8):1277–83. doi: 10.1017/S0007114510004812 21255470 10.1017/S0007114510004812

[10] Krug S, Kastenmuller G, Stuckler F, Rist MJ, Skurk T, Sailer M, et al The dynamic range of the human metabolome revealed by challenges. FASEB journal: official publication of the Federation of American Societies for Experimental Biology. 2012;26(6):2607–19. 22426117 10.1096/fj.11-198093

[11] Rheaume C, Arsenault BJ, Dumas MP, Perusse L, Tremblay A, Bouchard C, et al Contributions of cardiorespiratory fitness and visceral adiposity to six-year changes in cardiometabolic risk markers in apparently healthy men and women. J Clin Endocrinol Metab. 2011;96(5):1462–8. doi: 10.1210/jc.2010-2432 21325457 10.1210/jc.2010-2432

[12] Morris C, Grada CO, Ryan M, Roche HM, De Vito G, Gibney MJ, et al The relationship between aerobic fitness level and metabolic profiles in healthy adults. Molecular nutrition & food research. 2013;57(7):1246–54. 23505034 10.1002/mnfr.201200629

[13] Bye A, Vettukattil R, Aspenes ST, Giskeødegård GF, Gribbestad IS, Wisløff U, et al Serum Levels of Choline-Containing Compounds Are Associated with Aerobic Fitness Level: The HUNT-Study. PloS one. 2012;7(7): e42330 doi: 10.1371/journal.pone.0042330 22860113 10.1371/journal.pone.0042330 PMC3408491

[14] Newgard CB, An J, Bain JR, Muehlbauer MJ, Stevens RD, Lien LF, et al A branched-chain amino acid-related metabolic signature that differentiates obese and lean humans and contributes to insulin resistance. Cell Metab. 2009;9(4):311–26. PubMed Central PMCID: PMC3640280. doi: 10.1016/j.cmet.2009.02.002 19356713 10.1016/j.cmet.2009.02.002 PMC3640280

[15] Schulze MB, Hoffmann K, Boeing H, Linseisen J, Rohrmann S, Mohlig M, et al An accurate risk score based on anthropometric, dietary, and lifestyle factors to predict the development of type 2 diabetes. Diabetes Care. 2007;30(3):510–5. doi: 10.2337/dc06-2089 17327313 10.2337/dc06-2089

[16] Ford ES, Bergmann MM, Kroger J, Schienkiewitz A, Weikert C, Boeing H. Healthy living is the best revenge: findings from the European Prospective Investigation Into Cancer and Nutrition-Potsdam study. Archives of internal medicine. 2009;169(15):1355–62. doi: 10.1001/archinternmed.2009.237 19667296 10.1001/archinternmed.2009.237

[17] Lustgarten MS, Price LL, Phillips EM, Fielding RA. Serum glycine is associated with regional body fat and insulin resistance in functionally-limited older adults. PloS one. 2013;8(12):e84034 PubMed Central PMCID: PMC3877144. doi: 10.1371/journal.pone.0084034 24391874 10.1371/journal.pone.0084034 PMC3877144

[18] Martin FP, Montoliu I, Collino S, Scherer M, Guy P, Tavazzi I, et al Topographical body fat distribution links to amino acid and lipid metabolism in healthy obese women [corrected]. PLoS One. 2013;8(9):e73445 PubMed Central PMCID: PMC3770640. doi: 10.1371/journal.pone.0073445 24039943 10.1371/journal.pone.0073445 PMC3770640

[19] Jourdan C, Petersen AK, Gieger C, Doring A, Illig T, Wang-Sattler R, et al Body fat free mass is associated with the serum metabolite profile in a population-based study. PloS one. 2012;7(6):e40009 PubMed Central PMCID: PMC3384624. doi: 10.1371/journal.pone.0040009 22761945 10.1371/journal.pone.0040009 PMC3384624

[20] Floegel A, Stefan N, Yu Z, Muhlenbruch K, Drogan D, Joost HG, et al Identification of serum metabolites associated with risk of type 2 diabetes using a targeted metabolomic approach. Diabetes. 2013;62(2):639–48. PubMed Central PMCID: PMC3554384. doi: 10.2337/db12-0495 23043162 10.2337/db12-0495 PMC3554384

[21] Fox CS, Massaro JM, Hoffmann U, Pou KM, Maurovich-Horvat P, Liu CY, et al Abdominal visceral and subcutaneous adipose tissue compartments: association with metabolic risk factors in the Framingham Heart Study. Circulation. 2007;116(1):39–48. Epub 2007/06/20. doi: 10.1161/CIRCULATIONAHA.106.675355 17576866 10.1161/CIRCULATIONAHA.106.675355

[22] Kim SK, Kim HJ, Hur KY, Choi SH, Ahn CW, Lim SK, et al Visceral fat thickness measured by ultrasonography can estimate not only visceral obesity but also risks of cardiovascular and metabolic diseases. Am J Clin Nutr. 2004;79(4):593–9. Epub 2004/03/31. 15051602 10.1093/ajcn/79.4.593

[23] Bosy-Westphal A, Geisler C, Onur S, Korth O, Selberg O, Schrezenmeir J, et al Value of body fat mass vs anthropometric obesity indices in the assessment of metabolic risk factors. Int J Obes (Lond). 2006;30(3):475–83. 16261188 10.1038/sj.ijo.0803144

[24] Neeland IJ, Ayers CR, Rohatgi AK, Turer AT, Berry JD, Das SR, et al Associations of visceral and abdominal subcutaneous adipose tissue with markers of cardiac and metabolic risk in obese adults. Obesity (Silver Spring). 2013;21(9):E439–47. PubMed Central PMCID: PMC3751977. 23687099 10.1002/oby.20135 PMC3751977

[25] Booth A, Magnuson A, Foster M. Detrimental and protective fat: body fat distribution and its relation to metabolic disease. Hormone molecular biology and clinical investigation. 2014;17(1):13–27. doi: 10.1515/hmbci-2014-0009 25372727 10.1515/hmbci-2014-0009

[26] Bays HE, Fox KM, Grandy S, Group SS. Anthropometric measurements and diabetes mellitus: clues to the "pathogenic" and "protective" potential of adipose tissue. Metab Syndr Relat Disord. 2010;8(4):307–15. doi: 10.1089/met.2009.0089 20235743 10.1089/met.2009.0089

[27] Gronwald W, Klein MS, Kaspar H, Fagerer SR, Nurnberger N, Dettmer K, et al Urinary metabolite quantification employing 2D NMR spectroscopy. Anal Chem. 2008;80(23):9288–97. doi: 10.1021/ac801627c 19551947 10.1021/ac801627c

[28] Zacharias HU, Hochrein J, Klein MS, Samol C, Oefner PJ, Gronwald W. Current Experimental, Bioinformatic and Statistical Methods used in NMR Based Metabolomics. Current Metabolomics. 2013;1(3):253–68.

[29] Klein MS, Oefner PJ, Gronwald W. MetaboQuant: a tool combining individual peak calibration and outlier detection for accurate metabolite quantification in 1D (1)H and (1)H-(13)C HSQC NMR spectra. BioTechniques. 2013;54(5):251–6. doi: 10.2144/000114026 23662895 10.2144/000114026

[30] Wishart DS, Jewison T, Guo AC, Wilson M, Knox C, Liu Y, et al HMDB 3.0—The Human Metabolome Database in 2013. Nucleic acids research. 2013;41(Database issue):D801–D7. doi: 10.1093/nar/gks1065 23161693 10.1093/nar/gks1065 PMC3531200

[31] Schlecht I, Wiggermann P, Behrens G, Fischer B, Koch M, Freese J, et al Reproducibility and validity of ultrasound for the measurement of visceral and subcutaneous adipose tissues. Metabolism. 2014;63(12):1512–9. doi: 10.1016/j.metabol.2014.07.012 25242434 10.1016/j.metabol.2014.07.012

[32] Ainsworth BE, Haskell WL, Herrmann SD, Meckes N, Bassett DR Jr., Tudor-Locke C, et al 2011 Compendium of Physical Activities: a second update of codes and MET values. Med Sci Sports Exerc. 2011;43(8):1575–81. doi: 10.1249/MSS.0b013e31821ece12 21681120 10.1249/MSS.0b013e31821ece12

[33] Levey AS, Bosch JP, Lewis JB, Greene T, Rogers N, Roth D. A More Accurate Method To Estimate Glomerular Filtration Rate from Serum Creatinine: A New Prediction Equation. Annals of internal medicine. 1999;130(6):461–70. 10075613 10.7326/0003-4819-130-6-199903160-00002

[34] Rosner B. Fundamentals of Biostatistics. Belmont, CA: Brooks/Cole; 2011.

[35] Shapiro SS, Wilk MB. An Analysis of Variance Test for Normality (Complete Samples). Biometrika. 1965;52:591-&.

[36] Cloarec O, Dumas ME, Craig A, Barton RH, Trygg J, Hudson J, et al Statistical total correlation spectroscopy: an exploratory approach for latent biomarker identification from metabolic 1H NMR data sets. Anal Chem. 2005;77(5):1282–9. doi: 10.1021/ac048630x 15732908 10.1021/ac048630x

[37] Holmes E, Loo RL, Cloarec O, Coen M, Tang H, Maibaum E, et al Detection of urinary drug metabolite (xenometabolome) signatures in molecular epidemiology studies via statistical total correlation (NMR) spectroscopy. Anal Chem. 2007;79(7):2629–40. doi: 10.1021/ac062305n 17323917 10.1021/ac062305n PMC6688492

[38] Benjamini Y, Hochberg Y. Controlling the false discovery rate: A practical and powerful approach to multiple testing. J R Stat Soc B. 1995;57:289–300.

[39] Zeisel SH, da Costa KA. Choline: an essential nutrient for public health. Nutrition reviews. 2009;67(11):615–23. PubMed Central PMCID: PMC2782876. doi: 10.1111/j.1753-4887.2009.00246.x 19906248 10.1111/j.1753-4887.2009.00246.x PMC2782876

[40] Penry JT, Manore MM. Choline: an important micronutrient for maximal endurance-exercise performance? International journal of sport nutrition and exercise metabolism. 2008;18(2):191–203. 18458362 10.1123/ijsnem.18.2.191

[41] Zhang X, Wang Y, Hao F, Zhou X, Han X, Tang H, et al Human serum metabonomic analysis reveals progression axes for glucose intolerance and insulin resistance statuses. Journal of proteome research. 2009;8(11):5188–95. doi: 10.1021/pr900524z 19697961 10.1021/pr900524z

[42] Bodi V, Sanchis J, Morales JM, Marrachelli VG, Nunez J, Forteza MJ, et al Metabolomic profile of human myocardial ischemia by nuclear magnetic resonance spectroscopy of peripheral blood serum: a translational study based on transient coronary occlusion models. J Am Coll Cardiol. 2012;59(18):1629–41. doi: 10.1016/j.jacc.2011.09.083 22538333 10.1016/j.jacc.2011.09.083

[43] Detopoulou P, Panagiotakos DB, Antonopoulou S, Pitsavos C, Stefanadis C. Dietary choline and betaine intakes in relation to concentrations of inflammatory markers in healthy adults: the ATTICA study. Am J Clin Nutr. 2008;87(2):424–30. 18258634 10.1093/ajcn/87.2.424

[44] Oudi ME, Aouni Z, Mazigh C, Khochkar R, Gazoueni E, Haouela H, et al Homocysteine and markers of inflammation in acute coronary syndrome. Experimental and clinical cardiology. 2010;15(2):e25–8. Epub 2010/07/16. PubMed Central PMCID: PMCPMC2898531. 20631860 PMC2898531

[45] Elliott P, Posma JM, Chan Q, Garcia-Perez I, Wijeyesekera A, Bictash M, et al Urinary metabolic signatures of human adiposity. Science translational medicine. 2015;7(285):285ra62 doi: 10.1126/scitranslmed.aaa5680 25925681 10.1126/scitranslmed.aaa5680 PMC6598200

[46] Wang Z, Klipfell E, Bennett BJ, Koeth R, Levison BS, Dugar B, et al Gut flora metabolism of phosphatidylcholine promotes cardiovascular disease. Nature. 2011;472(7341):57–63. PubMed Central PMCID: PMC3086762. doi: 10.1038/nature09922 21475195 10.1038/nature09922 PMC3086762

[47] Hartiala J, Bennett BJ, Tang WH, Wang Z, Stewart AF, Roberts R, et al Comparative genome-wide association studies in mice and humans for trimethylamine N-oxide, a proatherogenic metabolite of choline and L-carnitine. Arterioscler Thromb Vasc Biol. 2014;34(6):1307–13. PubMed Central PMCID: PMC4035110. doi: 10.1161/ATVBAHA.114.303252 24675659 10.1161/ATVBAHA.114.303252 PMC4035110

[48] Dumas M-E, Barton RH, Toye A, Cloarec O, Blancher C, Rothwell A, et al Metabolic profiling reveals a contribution of gut microbiota to fatty liver phenotype in insulin-resistant mice. Proceedings of the National Academy of Sciences. 2006;103(33):12511–6. 10.1073/pnas.0601056103 PMC1567909 16895997

[49] DiBaise JK, Frank DN, Mathur R. Impact of the Gut Microbiota on the Development of Obesity: Current Concepts. Am J Gastroenterol Suppl. 2012;1(1):22–7.

[50] Brun P, Castagliuolo I, Di Leo V, Buda A, Pinzani M, Palu G, et al Increased intestinal permeability in obese mice: new evidence in the pathogenesis of nonalcoholic steatohepatitis. American journal of physiology Gastrointestinal and liver physiology. 2007;292(2):G518–25. Epub 2006/10/07. doi: 10.1152/ajpgi.00024.2006 17023554 10.1152/ajpgi.00024.2006

[51] Jacobs D, Spiesser L, Garnier M, de Roo N, van Dorsten F, Hollebrands B, et al SPE–NMR metabolite sub-profiling of urine. Analytical and Bioanalytical Chemistry. 2012;404(8):2349–61. doi: 10.1007/s00216-012-6339-2 22932811 10.1007/s00216-012-6339-2

[52] van Dorsten FA, Grün CH, van Velzen EJJ, Jacobs DM, Draijer R, van Duynhoven JPM. The metabolic fate of red wine and grape juice polyphenols in humans assessed by metabolomics. Molecular nutrition & food research. 2010;54(7):897–908. 20013882 10.1002/mnfr.200900212

[53] Butelli E, Titta L, Giorgio M, Mock H-P, Matros A, Peterek S, et al Enrichment of tomato fruit with health-promoting anthocyanins by expression of select transcription factors. Nat Biotech. 2008;26(11):1301–8. http://www.nature.com/nbt/journal/v26/n11/suppinfo/nbt.1506_S1.html. 10.1038/nbt.1506 18953354

[54] Tsuda T, Horio F, Uchida K, Aoki H, Osawa T. Dietary cyanidin 3-O-beta-D-glucoside-rich purple corn color prevents obesity and ameliorates hyperglycemia in mice. The Journal of nutrition. 2003;133(7):2125–30. Epub 2003/07/04. 12840166 10.1093/jn/133.7.2125

[55] Psychogios N, Hau DD, Peng J, Guo AC, Mandal R, Bouatra S, et al The human serum metabolome. PloS one. 2011;6(2):e16957 PubMed Central PMCID: PMC3040193. doi: 10.1371/journal.pone.0016957 21359215 10.1371/journal.pone.0016957 PMC3040193

[56] Bouatra S, Aziat F, Mandal R, Guo AC, Wilson MR, Knox C, et al The human urine metabolome. PloS one. 2013;8(9):e73076 PubMed Central PMCID: PMC3762851. doi: 10.1371/journal.pone.0073076 24023812 10.1371/journal.pone.0073076 PMC3762851

[57] Gowda GAN, Raftery D. Quantitating Metabolites in Protein Precipitated Serum Using NMR Spectroscopy. Analytical Chemistry. 2014;86(11):5433–40. doi: 10.1021/ac5005103 24796490 10.1021/ac5005103 PMC4045325

[58] Szymanska E, Bouwman J, Strassburg K, Vervoort J, Kangas AJ, Soininen P, et al Gender-dependent associations of metabolite profiles and body fat distribution in a healthy population with central obesity: towards metabolomics diagnostics. Omics: a journal of integrative biology. 2012;16(12):652–67. doi: 10.1089/omi.2012.0062 23215804 10.1089/omi.2012.0062

[59] Xie G, Ma X, Zhao A, Wang C, Zhang Y, Nieman D, et al The metabolite profiles of the obese population are gender-dependent. Journal of proteome research. 2014;13(9):4062–73. doi: 10.1021/pr500434s 25132568 10.1021/pr500434s PMC6098236

[60] Moore SC, Matthews CE, Sampson JN, Stolzenberg-Solomon RZ, Zheng W, Cai Q, et al Human metabolic correlates of body mass index. Metabolomics. 2014;10(2):259–69. PubMed Central PMCID: PMC4169991. doi: 10.1007/s11306-013-0574-1 25254000 10.1007/s11306-013-0574-1 PMC4169991

[61] Wang TJ, Larson MG, Vasan RS, Cheng S, Rhee EP, McCabe E, et al Metabolite profiles and the risk of developing diabetes. Nature medicine. 2011;17(4):448–53. Epub 2011/03/23. PubMed Central PMCID: PMCPMC3126616. doi: 10.1038/nm.2307 21423183 10.1038/nm.2307 PMC3126616

[62] Herman MA, She P, Peroni OD, Lynch CJ, Kahn BB. Adipose tissue branched chain amino acid (BCAA) metabolism modulates circulating BCAA levels. The Journal of biological chemistry. 2010;285(15):11348–56. Epub 2010/01/23. PubMed Central PMCID: PMCPMC2857013. doi: 10.1074/jbc.M109.075184 20093359 10.1074/jbc.M109.075184 PMC2857013

[63] Newgard CB. Interplay between lipids and branched-chain amino acids in development of insulin resistance. Cell Metab. 2012;15(5):606–14. Epub 2012/05/09. PubMed Central PMCID: PMCPMC3695706. doi: 10.1016/j.cmet.2012.01.024 22560213 10.1016/j.cmet.2012.01.024 PMC3695706

[64] Floegel A, Drogan D, Wang-Sattler R, Prehn C, Illig T, Adamski J, et al Reliability of serum metabolite concentrations over a 4-month period using a targeted metabolomic approach. PloS one. 2011;6(6):e21103 PubMed Central PMCID: PMC3115978. doi: 10.1371/journal.pone.0021103 21698256 10.1371/journal.pone.0021103 PMC3115978

