# Supplementary figures and images for: Visceral adipose tissue but not subcutaneous adipose tissue is associated with urine and serum metabolites
Source: PLoS One. 2017 Apr 12;12(4):e0175133. doi: 10.1371/journal.pone.0175133 (PMC5389790; doi:10.1371/journal.pone.0175133)

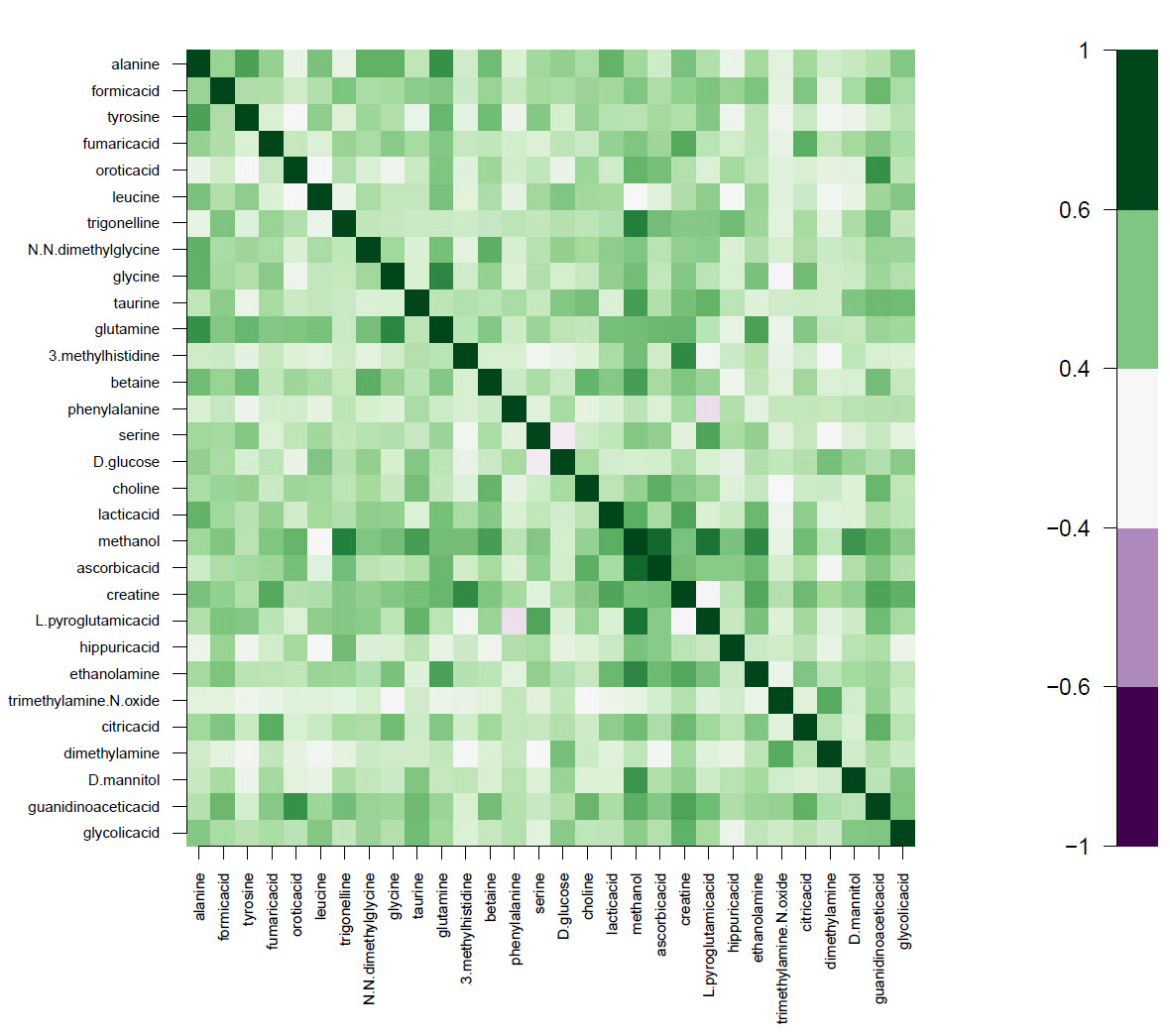

Supplement: S1 Fig — (JPG) [file pone.0175133.s001.jpg]

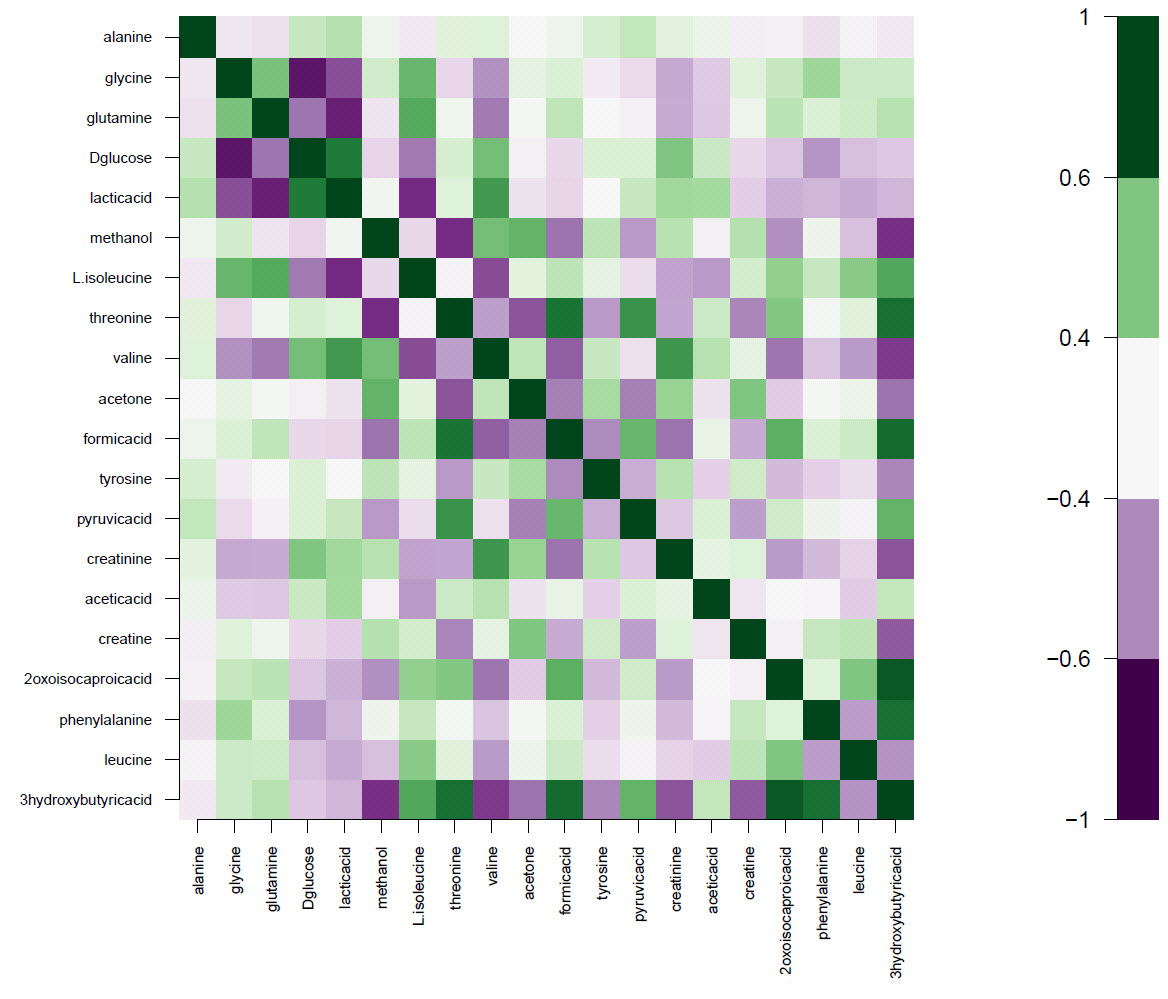

Supplement: S2 Fig — (JPG) [file pone.0175133.s002.jpg]

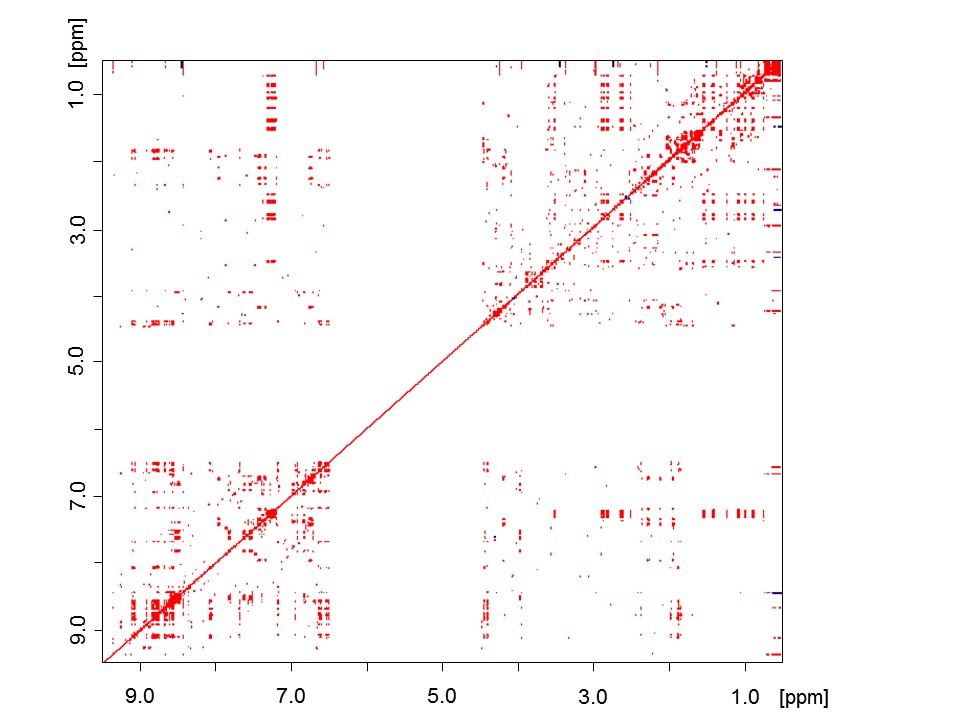

Supplement: S3 Fig — Positive and negative correlations are depicted in red and blue, respectively. Correlations above 0.50 respectively below -0.50 are shown. The water region and the region of the broad urea signal between 6.5–4.5 ppm was excluded. (JPG) [file pone.0175133.s003.jpg]
