# Supplementary material for: Visceral adipose tissue but not subcutaneous adipose tissue is associated with urine and serum metabolites
Source: PLoS One. 2017 Apr 12;12(4):e0175133. doi: 10.1371/journal.pone.0175133 (PMC5389790; doi:10.1371/journal.pone.0175133)
Supplement: S1 Table — All metabolites were quantified by 1D or 2D nuclear magnetic resonance spectroscopy: 1Metabolites quantified from 1D spectra, 2Metabolites quantified from 2D spectra, conc. = concentration, min = minimum, max = maximum, N = number of values above LLOQ. *Concentrations are influenced by Phenylalanine, Phenylacetylglycine und Phenylacetylglutamine. +Choline concentrations are likely to be influenced by other compounds including creatinine. (DOCX) [file pone.0175133.s004.docx]

Supplementary Table 1: Gender-dependent urinary metabolite concentrations.

| Metabolite  (µmol/mmol creatinine) | LLOQ | Total | | | Men | | | Women | | |
| --- | --- | --- | --- | --- | --- | --- | --- | --- | --- | --- |
|  |  | conc. range | | N/228 | conc. range | | N/107 | conc. range | | N/121 |
|  |  | min | max |  | min | max |  | min | max |  |
| ²Alanine | 0.078 | 6.52 | 76.70 | 227 | 6.52 | 76.70 | 106 | 6.91 | 70.73 | 121 |
| ²Glycine | 0.156 | 13.06 | 923.12 | 204 | 28.99 | 341.51 | 92 | 13.06 | 923.12 | 112 |
| ²Taurine | 0.312 | 17.44 | 324.20 | 88 | 17.44 | 186.10 | 60 | 18.71 | 324.20 | 28 |
| ²Glutamine | 0.312 | 19.10 | 98.87 | 80 | 19.83 | 82.58 | 49 | 19.10 | 98.87 | 31 |
| ²3-Methylhistidine | 0.078 | 7.25 | 650.17 | 125 | 7.25 | 406.68 | 68 | 9.84 | 650.17 | 57 |
| ²Betaine | 0.078 | 2.76 | 109.74 | 112 | 6.14 | 54.68 | 63 | 2.76 | 109.74 | 49 |
| ²Phenylcompound* | 0.141 | 17.12 | 416.80 | 154 | 17.12 | 416.80 | 71 | 21.16 | 274.71 | 83 |
| ²Serine | 0.281 | 13.18 | 323.41 | 65 | 27.77 | 323.41 | 42 | 13.18 | 139.48 | 23 |
| ²D-Glucose | 0.563 | 36.27 | 593.79 | 45 | 40.14 | 593.79 | 26 | 36.27 | 467.01 | 19 |
| ²Choline^+^ | 0.141 | 6.71 | 97.19 | 90 | 10.93 | 97.19 | 59 | 6.71 | 55.48 | 31 |
| ²Lactic acid | 0.078 | 5.94 | 954.05 | 97 | 9.04 | 512.95 | 44 | 5.94 | 954.05 | 53 |
| ²Methanol | 0.141 | 4.17 | 217.43 | 78 | 8.42 | 155.69 | 31 | 4.17 | 217.43 | 47 |
| ²Ascorbic acid | 0.312 | 13.15 | 1125.48 | 55 | 17.92 | 1125.48 | 27 | 13.15 | 569.02 | 28 |
| ²Creatine | 0.156 | 7.83 | 1131.94 | 68 | 7.83 | 391.44 | 22 | 11.07 | 1131.94 | 46 |
| ²L-Pyroglutamic acid | 0.312 | 12.98 | 106.57 | 32 | 16.48 | 59.15 | 16 | 12.98 | 106.57 | 16 |
| ²Hippuric acid | 0.312 | 20.02 | 1947.11 | 208 | 20.02 | 1467.01 | 93 | 25.43 | 1947.11 | 115 |
| ²Ethanolamine | 0.312 | 14.69 | 190.90 | 101 | 18.45 | 95.71 | 57 | 14.69 | 114.18 | 44 |
| ²Trimethylamine-N-oxide | 0.039 | 7.40 | 494.26 | 210 | 7.40 | 494.26 | 100 | 10.84 | 166.39 | 110 |
| ²Citricacid | 0.312 | 50.52 | 838.99 | 212 | 54.30 | 654.03 | 94 | 50.52 | 838.99 | 118 |
| ²Dimethylamine | 0.156 | 19.26 | 98.70 | 130 | 20.90 | 98.70 | 76 | 19.26 | 77.10 | 54 |
| ²D-Mannitol | 0.268 | 21.46 | 1138.04 | 130 | 21.46 | 1138.04 | 66 | 21.99 | 717.36 | 64 |
| ²Guanidinoacetic acid | 0.312 | 16.43 | 171.40 | 78 | 16.43 | 104.02 | 38 | 16.75 | 162.41 | 40 |
| ²Glycolic acid | 0.312 | 23.78 | 137.74 | 97 | 36.03 | 114.23 | 59 | 23.78 | 137.74 | 38 |
| ^1^Formic acid | 0.003 | 3.97 | 188.19 | 227 | 4.02 | 59.89 | 106 | 3.97 | 188.19 | 121 |
| ^1^Tyrosine | 0.002 | 0.82 | 31.53 | 196 | 2.09 | 31.53 | 95 | 0.82 | 24.85 | 101 |
| ^1^Fumaric acid | 0.002 | 0.08 | 4.36 | 107 | 0.08 | 1.94 | 50 | 0.13 | 4.36 | 57 |
| ^1^Orotic acid | 0.002 | 0.11 | 12.32 | 68 | 0.11 | 12.32 | 38 | 0.15 | 1.45 | 30 |
| ^1^Leucine | 0.002 | 0.54 | 11.56 | 217 | 0.54 | 7.35 | 106 | 1.13 | 11.56 | 114 |
| ^1^N,N-Dimethylglycine | 0.020 | 2.38 | 17.99 | 157 | 2.50 | 13.40 | 80 | 2.38 | 17.99 | 77 |
| ^1^Trigonelline | 0.020 | 0.87 | 187.33 | 196 | 0.87 | 133.54 | 90 | 1.11 | 187.33 | 106 |

All metabolites were quantified by 1D or 2D nuclear magnetic resonance spectroscopy: ^1^Metabolites quantified from 1D spectra, ²Metabolites quantified from 2D spectra,

conc.=concentration, min=minimum, max=maximum, N=number of values above LLOQ.

*Concentrations are influenced by Phenylalanine, Phenylacetylglycine und Phenylacetylglutamine.

^+^Choline concentrations are likely to be influenced by other compounds including creatinine.
