# Supplementary material for: Visceral adipose tissue but not subcutaneous adipose tissue is associated with urine and serum metabolites
Source: PLoS One. 2017 Apr 12;12(4):e0175133. doi: 10.1371/journal.pone.0175133 (PMC5389790; doi:10.1371/journal.pone.0175133)
Supplement: S2 Table — All metabolites were quantified by 1D or 2D nuclear magnetic resonance spectroscopy: 1Metabolites quantified from 1D spectra, 2Metabolites quantified from 2D spectra, conc. = concentration, min = minimum, max = maximum, N = number of available values. (DOCX) [file pone.0175133.s005.docx]

Supplementary Table 2: Gender-dependent serum metabolite concentration.

| Metabolite  (µmol/L) | LLOQ | Total | | | Men | | | Women | | |
| --- | --- | --- | --- | --- | --- | --- | --- | --- | --- | --- |
|  |  | conc. range | | N/200 | conc. range | | N/90 | conc. range | | N/110 |
|  |  | min | max |  | min | max |  | min | max |  |
| ²Alanine | 0.078 | 144.94 | 596.96 | 200 | 144.94 | 596.96 | 90 | 174.20 | 576.65 | 110 |
| ²Glycine | 0.156 | 157.63 | 535.34 | 157 | 158.13 | 535.34 | 71 | 157.63 | 445.24 | 86 |
| ²Glutamine | 0.312 | 316.92 | 800.05 | 186 | 322.97 | 718.42 | 81 | 316.92 | 800.05 | 105 |
| ²D-glucose | 0.563 | 2614.40 | 10283.96 | 200 | 2628.09 | 10006.38 | 90 | 2614.40 | 10283.96 | 110 |
| ²Lactic acid | 0.078 | 875.12 | 3865.71 | 200 | 937.85 | 3205.56 | 90 | 875.12 | 3865.71 | 110 |
| ²Methanol | 0.141 | 142.84 | 401.21 | 105 | 144.98 | 401.21 | 55 | 142.84 | 364.13 | 50 |
| ^1^L-isoleucine | 0.002 | 24.06 | 120.62 | 200 | 24.06 | 120.62 | 90 | 29.57 | 119.39 | 110 |
| ^1^Threonine | 0.002 | 26.28 | 205.32 | 200 | 47.30 | 200.00 | 90 | 26.28 | 205.32 | 110 |
| ^1^Valine | 0.002 | 118.01 | 348.86 | 200 | 118.01 | 346.28 | 90 | 146.22 | 348.86 | 110 |
| ^1^Acetone | 0.002 | 5.60 | 197.62 | 200 | 5.60 | 197.62 | 90 | 8.11 | 106.92 | 110 |
| ^1^Formic acid | 0.003 | 5.23 | 63.52 | 200 | 5.23 | 41.78 | 90 | 5.72 | 63.52 | 110 |
| ^1^Tyrosine | 0.002 | 23.81 | 123.39 | 200 | 26.03 | 123.39 | 90 | 23.81 | 109.13 | 110 |
| ^1^Pyruvic acid | 0.001 | 8.33 | 132.23 | 200 | 8.33 | 64.89 | 90 | 9.53 | 132.23 | 110 |
| ^1^Creatinine | 0.003 | 35.33 | 148.86 | 200 | 35.55 | 120.32 | 90 | 35.33 | 148.86 | 110 |
| ^1^Acetic acid | 0.003 | 7.79 | 87.69 | 200 | 8.96 | 87.69 | 90 | 7.79 | 83.55 | 110 |
| ^1^Creatine | 0.005 | 6.36 | 148.87 | 200 | 6.36 | 123.24 | 90 | 7.06 | 148.87 | 110 |
| ^1^Ketoleucin | 0.001 | 2.58 | 11.00 | 150 | 2.63 | 10.69 | 70 | 2.58 | 11.00 | 80 |
| ^1^Phenylalanine | 0.003 | 13.63 | 72.95 | 184 | 14.21 | 71.39 | 83 | 13.63 | 72.95 | 101 |
| ^1^Leucine | 0.003 | 45.78 | 202.29 | 200 | 45.78 | 202.29 | 90 | 55.84 | 189.02 | 110 |
| ^1^3-Hydroxybutyric acid | 0.002 | 9.48 | 978.88 | 189 | 9.48 | 978.88 | 87 | 10.42 | 329.56 | 102 |

All metabolites were quantified by 1D or 2D nuclear magnetic resonance spectroscopy: ^1^Metabolites quantified from 1D spectra, ²Metabolites quantified from 2D spectra, conc.=concentration, min=minimum, max=maximum, N=number of available values.
