# Supplementary material for: Visceral adipose tissue but not subcutaneous adipose tissue is associated with urine and serum metabolites
Source: PLoS One. 2017 Apr 12;12(4):e0175133. doi: 10.1371/journal.pone.0175133 (PMC5389790; doi:10.1371/journal.pone.0175133)
Supplement: S3 Table — P-value from Kruskal-Wallis test. (DOCX) [file pone.0175133.s006.docx]

Supplementary Table 3: Urinary metabolite interaction with sex, fasting status, and urinary glucose.

|  | Sex  p value | Fasting status  p value | Urinary glucose  p value |
| --- | --- | --- | --- |
| Alanine | 0.142 | 0.002 | 0.548 |
| Glycine | <0.001 | 0.125 | 0.056 |
| Taurine | 0.795 | 0.780 | 0.006 |
| Glutamine | 0.204 | 0.013 | 0.246 |
| 3-methylhistidine | 0.024 | 0.218 | 0.003 |
| Betaine | 0.413 | 0.078 | 0.028 |
| Phenylalanine | <0.001 | 0.668 | 0.041 |
| Serine | 0.459 | 0.146 | 0.007 |
| D-glucose | 0.301 | 0.252 | - |
| Choline | 0.302 | 0.768 | 0.017 |
| Lactic acid | <0.001 | 0.180 | 0.005 |
| Methanol | 0.007 | 0.003 | <0.001 |
| Ascorbic acid | 0.096 | 0.009 | <0.001 |
| Creatine | 0.001 | 0.165 | <0.001 |
| L-pyroglutamic acid | 0.546 | 0.024 | 0.001 |
| Hippuricacid | <0.001 | 0.043 | <0.001 |
| Ethanolamine | 0.019 | 0.463 | 0.001 |
| Trimethylamine-N-oxide | 0.026 | 0.401 | 0.075 |
| Citric acid | <0.001 | 0.032 | 0.013 |
| Dimethylamine | <0.001 | 0.412 | 0.133 |
| D-mannitol | 0.002 | 0.037 | 0.001 |
| Guanidinoacetic acid | <0.001 | 0.048 | <0.001 |
| Glycolic acid | 0.209 | 0.001 | 0.659 |
| Formic acid | <0.001 | 0.004 | <0.001 |
| Tyrosine | 0.582 | 0.026 | 0.295 |
| Fumaric acid | <0.001 | 0.061 | 0.853 |
| Orotic acid | 0.217 | 0.442 | 0.002 |
| Leucine | 0.112 | 0.325 | 0.668 |
| N,N-dimethylglycine | 0.013 | 0.083 | 0.070 |
| Trigonelline | <0.001 | <0.001 | <0.001 |

P-value from Kruskal-Wallis test.
