# Supplementary material for: Visceral adipose tissue but not subcutaneous adipose tissue is associated with urine and serum metabolites
Source: PLoS One. 2017 Apr 12;12(4):e0175133. doi: 10.1371/journal.pone.0175133 (PMC5389790; doi:10.1371/journal.pone.0175133)
Supplement: S4 Table — P-value from Kruskal-Wallis test. (DOCX) [file pone.0175133.s007.docx]

Supplementary Table 4: Serum metabolite interaction with sex, fasting status, and urinary glucose.

|  | Sex  p value | Fasting status  p value | Urinary glucose  p value |
| --- | --- | --- | --- |
| Alanine | 0.412 | 0.357 | 0.115 |
| Glycine | 0.659 | 0.145 | 0.337 |
| Glutamine | 0.934 | 0.514 | 0.705 |
| D-glucose | 0.854 | 0.776 | 0.015 |
| Lacticacid | 0.352 | 0.466 | 0.029 |
| Methanol | 0.568 | 0.374 | 0.291 |
| L-isoleucine | 0.593 | 0.051 | 0.785 |
| Threonine | 0.824 | 0.016 | 0.248 |
| Valine | 0.894 | 0.038 | 0.741 |
| Acetone | 0.396 | 0.430 | 0.317 |
| Formic acid | 0.737 | 0.188 | 0.950 |
| Tyrosine | 0.850 | 0.018 | 0.480 |
| Pyruvic acid | 0.784 | 0.247 | 0.007 |
| Creatinine | 0.042 | 0.024 | 0.118 |
| Acetic acid | 0.688 | 0.939 | 0.227 |
| Creatine | 0.234 | 0.800 | 0.948 |
| Ketoleucine | 0.999 | 0.098 | 0.058 |
| Phenylalanine | 0.909 | 0.010 | 0.145 |
| Leucine | 0.410 | 0.055 | 0.712 |
| 3-hydroxybutyricacid | 0.856 | 0.924 | 0.256 |

P-value from Kruskal-Wallis test.
