# Supplementary material for: Visceral adipose tissue but not subcutaneous adipose tissue is associated with urine and serum metabolites
Source: PLoS One. 2017 Apr 12;12(4):e0175133. doi: 10.1371/journal.pone.0175133 (PMC5389790; doi:10.1371/journal.pone.0175133)
Supplement: S5 Table — Model 1: regression model adjusted for study, age (non-linear) and sex. Model 2: regression model adjusted for study, age and sex interaction (non-linear), smoking status, menopausal status (women only), physical activity, urinary glucose, and eGFR. VAT = visceral adipose tissue, BMI = body mass index, WC = waist circumference, ß = beta coefficient, p-value = corrected for multiple testing by controlling the false discovery rate. *linear model applied, p-values<0.05 after correction for multiple testing were considered significant. (DOCX) [file pone.0175133.s008.docx]

S5 Table: Significant associations between measures of obesity and urinary metabolite levels among subgroups.

|  | Metabolite | Model 1 | | Model 2 | |  |
| --- | --- | --- | --- | --- | --- | --- |
|  |  | ß | p | ß | p |  |
| **Non-fasting subjects (n=198)** | | | | | | |
| VAT | Formic acid* | -0.11 | 6.63*10^-5^ | -0.09 | 0.04 |  |
|  | Choline | -0.20 | 6.68*10^-6^ | -0.17 | 1.84*10^-4^ |  |
|  | Dimethylamine | 0.24 | 0.03 | 0.43 | 0.02 |  |
|  | Ethanolamine | 0.20 | 0.03 | 0.36 | 0.02 |  |
|  |  |  |  |  |  |  |
| BMI | Formic acid* | -0.05 | 0.01 | -0.06 | 0.09 |  |
|  | Choline | -0.10 | 0.04 | -0.19 | 0.02 |  |
|  | Creatine | -0.11 | 0.02 | -0.12 | 0.08 |  |
|  | Dimethylamine | 0.17 | 0.01 | 0.25 | 0.01 |  |
|  | Ethanolamine | 0.13 | 0.01 | 0.19 | 0.02 |  |
|  | Glutamine | -0.14 | 0.01 | -0.19 | 0.01 |  |
|  | Glycolic acid | -0.14 | 0.01 | -0.18 | 0.02 |  |
|  | Guanidinoacetic acid | -0.13 | 0.01 | -0.15 | 0.03 |  |
|  | Lactic acid | -0.09 | 0.05 | -0.21 | 0.01 |  |
|  | Methanol | 0.13 | 0.04 | 0.04 | 0.46 |  |
|  |  |  |  |  |  |  |
| WC | Formic acid* | -0.02 | 0.01 | -0.01 | 0.12 |  |
|  | Alanine | 0.03 | 0.03 | 0.06 | 0.06 |  |
|  | Betaine | -0.04 | 0.02 | -0.05 | 0.02 |  |
|  | Choline | -0.04 | 0.02 | -0.08 | 0.01 |  |
|  | Creatine | -0.04 | 0.03 | -0.03 | 0.21 |  |
|  | Dimethylamine | 0.07 | 1.84*10^-3^ | 0.09 | 0.01 |  |
|  | Ethanolamine | 0.05 | 4.72*10^-3^ | 0.07 | 0.01 |  |
|  | Glutamine | -0.05 | 4.72*10^-3^ | -0.07 | 0.01 |  |
|  | Glycolic acid | -0.04 | 0.02 | -0.06 | 0.01 |  |
|  | Guanidinoacetic acid | -0.05 | 4.72*10^-3^ | -0.06 | 0.01 |  |
|  | Lactic acid | -0.03 | 0.04 | -0.07 | 0.01 |  |
|  | Methanol | 0.04 | 0.04 | 0.03 | 0.22 |  |
|  | Serine | -0.04 | 0.03 | -0.07 | 0.01 |  |
| **Non-glucose subjects (n=183)** | | | | | | |
| VAT | Choline | -0.26 | 8.21*10^-7^ | -0.24 | 9.34*10^-5^ |  |
|  | Dimethylamine | 0.61 | 0.05 | 0.64 | 0.03 |  |
|  | Ethanolamine | 0.60 | 0.05 | 0.29 | 0.01 |  |
|  |  |  |  |  |  |  |
| BMI | Alanine | -0.10 | 0.03 | -0.15 | 0.06 |  |
|  | Creatine | -0.12 | 0.03 | -0.11 | 0.19 |  |
|  | Dimethylamine | 0.17 | 1.86*10^-3^ | 0.27 | 0.01 |  |
|  | Ethanolamine | 0.15 | 4.02*10^-3^ | 0.20 | 0.02 |  |
|  | Glutamine | -0.14 | 0.02 | -0.22 | 0.01 |  |
|  | Glycolicacid | -0.19 | 1.85*10^-3^ | -0.25 | 0.01 |  |
|  |  |  |  |  |  |  |
| WC | Alanine | 0.04 | 0.02 | 0.07 | 0.08 |  |
|  | Betaine | -0.04 | 0.04 | -0.04 | 0.12 |  |
|  | Choline | -0.05 | 0.04 | -0.09 | 0.05 |  |
|  | Creatine | -0.04 | 0.05 | -0.03 | 0.28 |  |
|  | Dimethylamine | 0.07 | 7.85*10^-$^ | 0.11 | 0.01 |  |
|  | Ethanolamine | 0.06 | 2.48*10^-3^ | 0.08 | 0.02 |  |
|  | Glutamine | -0.05 | 0.02 | -0.08 | 0.02 |  |
|  | Glycolic acid | -0.06 | 3.45*10^-3^ | -0.07 | 0.02 |  |
|  | Guanidinoacetic acid | -0.04 | 0.04 | -0.05 | 0.08 |  |
|  | Taurine | -0.04 | 0.05 | -0.04 | 0.20 |  |

Model 1: regression model adjusted for study, age (non-linear) and sex.

Model 2: regression model adjusted for study, age and sex interaction (non-linear), smoking status, menopausal status (women only), physical activity, urinary glucose, and eGFR.

VAT=visceral adipose tissue, BMI=body mass index, WC=waist circumference, ß=beta coefficient, p-value=corrected for multiple testing by controlling the false discovery rate.

*linear model applied, p-values<0.05 after correction for multiple testing were considered significant.
