# Supplementary material for: Visceral adipose tissue but not subcutaneous adipose tissue is associated with urine and serum metabolites
Source: PLoS One. 2017 Apr 12;12(4):e0175133. doi: 10.1371/journal.pone.0175133 (PMC5389790; doi:10.1371/journal.pone.0175133)
Supplement: S6 Table — Model 1: linear regression model adjusted for study, age (non-linear) and sex. Model 2: linear regression model adjusted for study, age and sex interaction (non-linear), smoking status, menopausal status (women only), physical activity, urinary glucose, and eGFR. In each case, the stratification variable was excluded from the model. VAT = visceral adipose tissue, ß = beta coefficient, p-value = corrected for multiple testing by controlling the false discovery rate. p-values<0.05 after correction for multiple testing were considered significant. (DOCX) [file pone.0175133.s009.docx]

Supplementary Table 5: Significant results from regression analyses to detect relations of VAT to urinary bins among subgroups.

|  |  | Model 1 | | Model 2 | |  | |
| --- | --- | --- | --- | --- | --- | --- | --- |
| Anthropometric variable | bin (ppm) | ß | p | ß | p | metabolite identification | |
| **Non-fasting subjects (n=198)** | | | | | | | |
| VAT | 9.135 | -0.153 | 0.0029 | -0.160 | 0.0020 | unknown | |
|  | 9.075 | -0.126 | 0.0076 | -0.128 | 0.0068 | unknown | |
|  | 8.455 | -0.089 | 0.0013 | -0.095 | 0.0005 | unknown | |
|  | 7.075 | -0.083 | 0.0036 | -0.083 | 0.0037 | 2,5-Furandicarboxylic acid, unknown | |
|  | 6.965 | -0.074 | 0.0016 | -0.078 | 0.0009 | 4-Hydroxyhippuric acid | |
|  | 6.955 | -0.065 | 0.0022 | -0.070 | 0.0008 | 4-Hydroxyhippuric acid | |
|  | 6.585 | -0.068 | 0.0005 | -0.072 | 0.0002 | unknown | |
|  | 6.575 | -0.056 | 0.0089 | -0.061 | 0.0038 | unknown | |
|  | 6.555 | -0.057 | 0.0093 | -0.062 | 0.0046 | unknown | |
|  | 4.095 | -0.036 | 0.0118 | -0.039 | 0.0059 | D-Saccharic acid | |
|  | 4.075 | -0.131 | 0.0011 | -0.133 | 0.0010 | Creatinine | |
|  | 4.065 | -0.118 | 0.0019 | -0.119 | 0.0019 | Choline/creatinine/others | |
|  | 3.945 | -0.044 | 0.0064 | -0.047 | 0.0034 | 4-Hydroxyhippuric acid/others | |
|  | 3.925 | -0.047 | 0.0024 | -0.050 | 0.0009 | Caffeine, tyrosine | |
|  | 3.565 | -0.086 | 0.0001 | -0.088 | 0.00004 | Glycine, sucrose |  |
|  | 3.525 | -0.056 | 0.0004 | -0.058 | 0.0002 | Choline/others |  |
|  | 3.365 | -0.095 | 0.0001 | -0.096 | 0.0001 | scyllo-Inositol | |
|  | 3.355 | -0.052 | 0.0008 | -0.053 | 0.0006 | scyllo-Inositol | |
|  | 3.345 | -0.062 | 0.0017 | -0.066 | 0.0007 | 3-Methylhistidine | |
|  | 3.205 | -0.055 | 0.0041 | -0.055 | 0.0044 | Choline/others | |
|  | 3.155 | -0.032 | 0.0073 | -0.032 | 0.0063 | 1-Methylhistidine | |
|  | 3.145 | -0.032 | 0.0039 | -0.033 | 0.0027 | cis-Aconitic acid | |
|  | 3.115 | -0.070 | 0.0057 | -0.070 | 0.0061 | 3-Aminoisobutyric acid | |
|  | 3.055 | 0.089 | 0.0069 | 0.093 | 0.0047 | Tyrosine/creatinine | |
|  | 2.985 | -0.030 | 0.0137 | -0.033 | 0.0064 | unknown | |
| VAT | 2.975 | -0.036 | 0.0047 | -0.040 | 0.0018 | Asparagine | |
|  | 2.965 | -0.037 | 0.0058 | -0.041 | 0.0024 | Asparagine |  |
|  | 2.835 | -0.038 | 0.0003 | -0.040 | 0.0001 | Methylguanidine |  |
|  | 2.775 | -0.025 | 0.0145 | -0.028 | 0.0046 | unknown |  |
|  | 2.735 | 0.068 | 0.0047 | 0.067 | 0.0054 | Dimethylamine |  |
|  | 2.725 | 0.061 | 0.0004 | 0.063 | 0.0003 | Dimethylamine/citric acid |  |
|  | 2.705 | -0.102 | 0.0061 | -0.103 | 0.0061 | Citric acid |  |
|  | 2.675 | -0.131 | 0.0011 | -0.133 | 0.0010 | Citric acid |  |
|  | 2.645 | -0.040 | 0.0017 | -0.043 | 0.0006 | unknown |  |
|  | 2.605 | -0.038 | 0.0010 | -0.038 | 0.0009 | Ketoleucine |  |
|  | 2.515 | -0.051 | 0.0001 | -0.053 | 0.00003 | L-Pyroglutamic acid |  |
|  | 2.475 | -0.041 | 0.0008 | -0.043 | 0.0004 | Pyridoxine, isocitric acid |  |
|  | 2.465 | -0.031 | 0.0029 | -0.033 | 0.0017 | 3-hydroxy-3-methylglutaric acid |  |
|  | 2.455 | -0.032 | 0.0016 | -0.033 | 0.0012 | Glutamine |  |
|  | 2.445 | -0.040 | 0.0003 | -0.041 | 0.0002 | Carnitine |  |
|  | 2.435 | -0.040 | 0.0001 | -0.042 | 0.0001 | 3-Hydroxy-3-methylglutaric acid |  |
|  | 2.425 | -0.036 | 0.0027 | -0.039 | 0.0008 | unknown |  |
|  | 2.405 | -0.051 | 0.0007 | -0.054 | 0.0002 | L-pyroglutamic acid |  |
|  | 2.395 | -0.029 | 0.0139 | -0.033 | 0.0041 | unknown |  |
|  | 2.365 | -0.040 | 0.0016 | -0.042 | 0.0006 | 3-Hydroxyisovaleric acid |  |
|  | 2.355 | -0.033 | 0.0050 | -0.036 | 0.0028 | unknown |  |
|  | 2.335 | -0.028 | 0.0105 | -0.031 | 0.0046 | unknown |  |
|  | 2.325 | -0.035 | 0.0016 | -0.038 | 0.0005 | unknown |  |
|  | 2.315 | -0.036 | 0.0021 | -0.039 | 0.0009 | unknown |  |
|  | 2.305 | -0.039 | 0.0055 | -0.041 | 0.0034 | unknown |  |
|  | 2.245 | -0.030 | 0.0058 | -0.033 | 0.0024 | 5-Aminopentanonic acid |  |
|  | 2.235 | -0.084 | 0.0000 | -0.086 | 0.00001 | Acetone |  |
| VAT | 2.225 | -0.030 | 0.0119 | -0.033 | 0.0058 | unknown |  |
|  | 2.215 | -0.034 | 0.0067 | -0.036 | 0.0036 | unknown |  |
|  | 2.205 | -0.036 | 0.0080 | -0.038 | 0.0053 | unknown |  |
|  | 2.195 | -0.042 | 0.0045 | -0.044 | 0.0030 | Suberic acid, sebacic acid |  |
|  | 2.185 | -0.058 | 0.0017 | -0.061 | 0.0010 | unknown |  |
|  | 2.175 | -0.044 | 0.0076 | -0.046 | 0.0047 | unknown |  |
|  | 2.155 | -0.032 | 0.0087 | -0.034 | 0.0069 | unknown, Hydroxyacetone |  |
|  | 2.135 | -0.035 | 0.0037 | -0.036 | 0.0027 | unknown |  |
|  | 2.125 | -0.039 | 0.0051 | -0.040 | 0.0052 | N-Acetyl-L-glutamine |  |
|  | 2.075 | -0.030 | 0.0115 | -0.032 | 0.0056 | unknown |  |
|  | 2.025 | -0.030 | 0.0046 | -0.032 | 0.0014 | L-Pyroglutamic acid |  |
|  | 2.015 | -0.030 | 0.0074 | -0.033 | 0.0026 | unknown |  |
|  | 2.005 | -0.034 | 0.0059 | -0.037 | 0.0020 | unknown |  |
|  | 1.815 | -0.033 | 0.0149 | -0.036 | 0.0062 | 4-Guanidinobutyric acid |  |
|  | 1.655 | -0.035 | 0.0103 | -0.038 | 0.0041 | Arginine |  |
|  | 1.645 | -0.036 | 0.0028 | -0.039 | 0.0012 | 5-Aminopentanoic acid, glycyl-L-leucine |  |
|  | 1.635 | -0.029 | 0.0089 | -0.031 | 0.0038 | 2-Aminoadipic acid |  |
|  | 1.625 | -0.029 | 0.0118 | -0.031 | 0.0055 | unknown |  |
|  | 1.615 | -0.028 | 0.0146 | -0.031 | 0.0071 | unknown |  |
|  | 1.555 | -0.033 | 0.0046 | -0.035 | 0.0026 | Suberic acid, sebacic acid |  |
|  | 1.475 | -0.036 | 0.0067 | -0.037 | 0.0066 | Lysine |  |
|  | 1.355 | 0.032 | 0.0136 | 0.035 | 0.0070 | unknown |  |
|  | 1.235 | -0.046 | 0.0021 | -0.049 | 0.0009 | unknown |  |
|  | 1.225 | -0.038 | 0.0095 | -0.041 | 0.0047 | unknown |  |
|  | 1.185 | -0.043 | 0.0073 | -0.046 | 0.0039 | unknown |  |
|  | 1.145 | -0.052 | 0.0104 | -0.058 | 0.0039 | Isobutyrylcarnitine |  |
|  | 1.095 | -0.039 | 0.0103 | -0.042 | 0.0050 | unknown |  |
|  | 1.015 | -0.032 | 0.0155 | -0.035 | 0.0070 | unknown |  |
| **Non-glucose subjects** | | | | | | |  |
| VAT | 2.405 | -0.052 | 0.0017 | -0.055 | 0.0448 | L-pyroglutamic acid |  |
|  | 2.435 | -0.033 | 0.0052 | -0.036 | 0.0011 | 3-Hydroxy-3-methylglutaric acid |  |
|  | 2.605 | -0.036 | 0.0039 | -0.039 | 0.0010 | Ketoleucine |  |
|  | 2.615 | -0.049 | 0.0043 | -0.056 | 0.0006 | Ketoleucine |  |
|  | 2.465 | -0.033 | 0.0052 | -0.037 | 0.0009 | 3-Hydroxy-3-methylglutaric acid |  |
|  | 3.345 | -0.057 | 0.0089 | -0.070 | 0.0007 | 3-Methylhistidine |  |
|  | 3.355 | -0.050 | 0.0030 | -0.055 | 0.0007 | scyllo-Inositol |  |
|  | 6.585 | -0.056 | 0.0063 | -0.064 | 0.0010 | unknown |  |
|  | 6.965 | -0.078 | 0.0037 | -0.086 | 0.0008 | 4-Hydroxyhippuric acid |  |
|  | 8.455 | -0.093 | 0.0014 | -0.091 | 0.0009 | unknown |  |
|  | 9.135 | -0.154 | 0.0036 | -0.162 | 0.0010 | unknown |  |

Model 1: linear regression model adjusted for study, age (non-linear) and sex.

Model 2: linear regression model adjusted for study, age and sex interaction (non-linear), smoking status, menopausal status (women only), physical activity, urinary glucose, and eGFR. In each case, the stratification variable was excluded from the model.

VAT=visceral adipose tissue, ß=beta coefficient, p-value=corrected for multiple testing by controlling the false discovery rate.

p-values<0.05 after correction for multiple testing were considered significant.
