# Supplementary material for: Visceral adipose tissue but not subcutaneous adipose tissue is associated with urine and serum metabolites
Source: PLoS One. 2017 Apr 12;12(4):e0175133. doi: 10.1371/journal.pone.0175133 (PMC5389790; doi:10.1371/journal.pone.0175133)
Supplement: S7 Table — Model 1: linear regression model adjusted for study, age (non-linear) and sex. Model 2: linear regression model adjusted for study, age and sex interaction (non-linear), smoking status, menopausal status (women only), physical activity, urinary glucose, and eGFR. In each case, the stratification variable was excluded from the model. VAT = visceral adipose tissue, ß = beta coefficient, p-value = corrected for multiple testing by controlling the false discovery rate. p-values<0.05 after correction for multiple testing were considered significant. (DOCX) [file pone.0175133.s010.docx]

S7 Table: Significant results from multiple regression analyses on the relation between VAT and serum bins among subgroups.

|  |  | Model 1 | | Model 2 | |  |
| --- | --- | --- | --- | --- | --- | --- |
| Anthropometric variable | bin (ppm) | ß | p | ß | p | metabolite identification |
| **Non-fasting men (n=78)** | | | | | | |
| VAT | 0.995 | 0.0413 | 0.0146 | 0.0588 | 0.0015 | Valine |
|  | 0.985 | 0.0381 | 0.0476 | 0.0138 | 0.0285 | Valine |
| VAT | 0.935 | 0.0210 | 0.5806 | 0.0454 | 0.0309 | Isoleucine, ketoleucine |
|  | 1.035 | 0.0694 | 0.0993 | 0.0962 | 0.0020 | unknown |
|  | 1.075 | 0.0494 | 0.2005 | 0.0764 | 0.0034 | Isobutyric acid, unknown |
|  | 1.085 | 0.0399 | 0.3770 | 0.0594 | 0.0467 | unknown |
|  | 1.105 | 0.0311 | 0.3770 | 0.0507 | 0.0219 | unknown |
|  | 2.595 | 0.0409 | 0.3770 | 0.0548 | 0.0467 | unknown |
|  | 2.605 | 0.0405 | 0.1133 | 0.0505 | 0.0136 | Ketoleucine |
|  | 2.615 | 0.0410 | 0.1447 | 0.0606 | 0.0085 | Ketoleucine |
|  | 2.625 | 0.0321 | 0.3385 | 0.0486 | 0.0034 | Ketoleucine |
|  | 2.635 | 0.0358 | 0.1447 | 0.0571 | 0.0006 | unknown |
|  | 2.655 | 0.0282 | 0.3384 | 0.0584 | 0.0023 | Methionine, unknown |
|  | 4.345 | -0.2383 | 0.2005 | -0.3690 | 0.0125 | unknown |
|  | 6.535 | 0.4483 | 0.3770 | 0.7294 | 0.0467 | unknown |
|  | 7.375 | 0.0356 | 0.4929 | 0.0761 | 0.0134 | Phenylalanine, unknown |
|  | 7.675 | -0.2247 | 0.3932 | -0.3475 | 0.0117 | unknown |
| **Non-fasting women (n=94)** | | | | | | |
| VAT | 0.605 | -0.4988 | 0.1530 | -0.4837 | 0.0033 | unknown |
|  | 0.735 | -0.0341 | 0.5236 | -0.0631 | 0.0049 | Lipid-cholesterol |
|  | 7.845 | -0.2551 | 0.3720 | -0.5066 | 0.0373 | unknown |

Model 1: linear regression model adjusted for study, age (non-linear) and sex.

Model 2: linear regression model adjusted for study, age and sex interaction (non-linear), smoking status, menopausal status (women only), physical activity, urinary glucose, and eGFR. In each case, the stratification variable was excluded from the model.

VAT=visceral adipose tissue, ß=beta coefficient, p-value=corrected for multiple testing by controlling the false discovery rate.

p-values<0.05 after correction for multiple testing were considered significant.
